# Supplementary material for: Human acute inflammatory recovery is defined by co-regulatory dynamics of white blood cell and platelet populations
Source: Nat Commun. 2022 Aug 22;13:4705. doi: 10.1038/s41467-022-32222-2 (PMC9395541; doi:10.1038/s41467-022-32222-2)
Supplement: Supplementary file 1 — Supplementary Information [file 41467_2022_32222_MOESM1_ESM.pdf]

Human Acute Inflammatory Recovery is Defined by

Co-Regulatory Dynamics of White Blood Cell and Platelet Populations

SUPPLEMENTARY FILE 1

Authors: Brody H Foy[1,2,3], Thor Sundt[4], Jonathan C T Carlson[1,5], Aaron D Aguirre[1, 6, 7], John M Higgins[1,2,3]

Affiliations:
[1]Center for Systems Biology, Massachusetts General Hospital, Harvard Medical School, Boston, USA
[2]Department of Pathology, Massachusetts General Hospital, Harvard Medical School, Boston, USA
[3]Department of Systems Biology, Harvard Medical School, Boston, USA
[4]Division of Cardiac Surgery, Corrigan Minehan Heart Center, Massachusetts General Hospital, Boston, USA
[5]Cancer Center, Massachusetts General Hospital, Harvard Medical School, Boston, USA
[6]Cardiology Division, Massachusetts General Hospital, Harvard Medical School, Boston, USA
[7]Wellman Center for Photomedicine, Massachusetts General Hospital, Harvard Medical School, Boston, USA

Corresponding authors: John M Higgins (john_higgins@hms.harvard.edu), Jonathan CT Carlson (carlson.jonathan@mgh.harvard.edu) and Aaron D Aguirre (aaguirre1@mgh.harvard.edu). Correspondence address: Simches Research Center, 185 Cambridge Street, Boston, MA, 02114.

Contents:

Supplementary Figures S1-S22

Supplementary Tables S1-S9

Supplementary References

Supplementary Figures

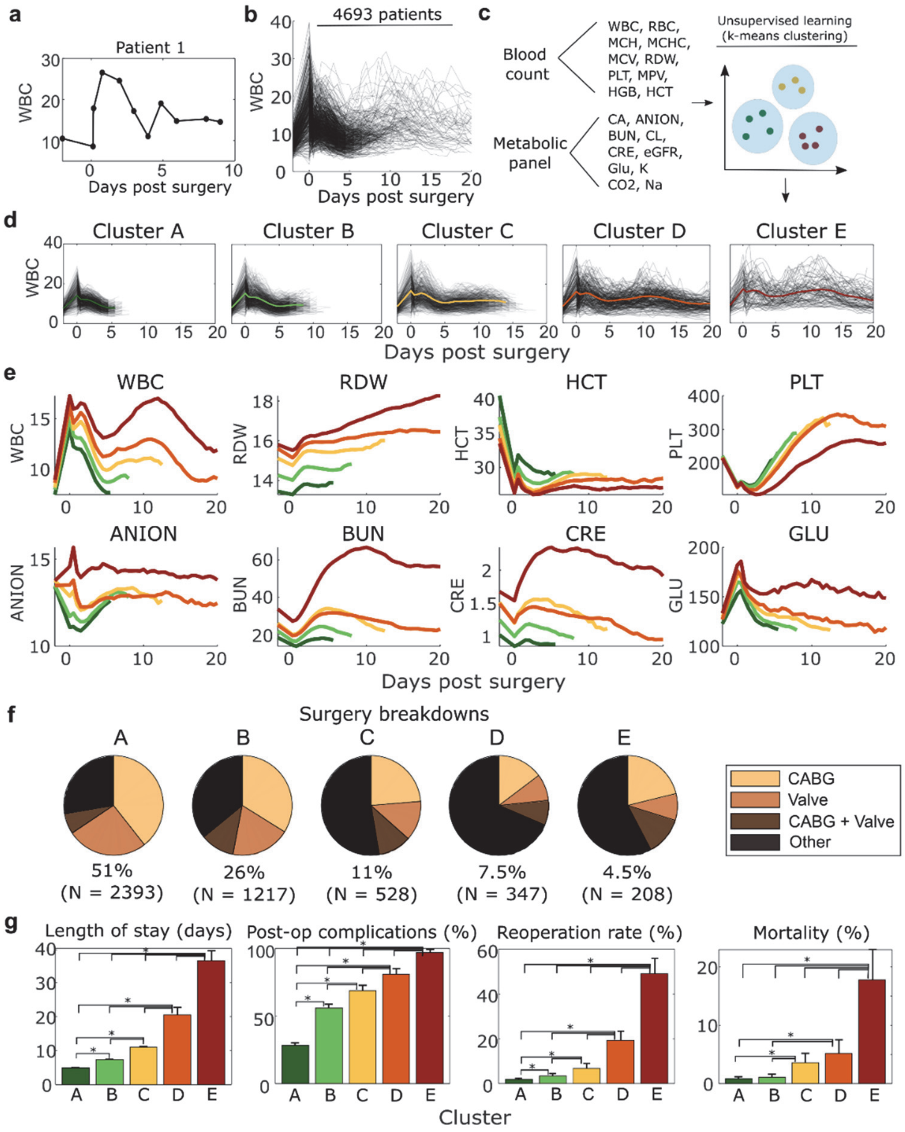

Supplementary Fig. 1 | High-dimensional clusters of response to cardiac surgery defined from routine clinical laboratory tests. Individual lab test results were interpolated (**a**), and these individual test trajectories (**b**) were considered for 20 standard complete blood count (CBC), basic metabolic panel (BMP), and other clinical laboratory tests (**c**). K-means clustering identified five distinct clusters (**d, e**), associated with significant differences (p<0.05) in surgery type (**f**) and post-operative outcomes (**g**). Day 0 corresponds to the first blood count measurement post-surgery, while day -2 corresponds to pre-operative measurements. Star (*) denotes values in (**g**) that are statistically significantly different (p > 0.05) from each other, using two-sided t-tests (for length of stay) and two-sided chi-squared tests (for complication, reoperation and mortality rates), without adjustment for multiple comparisons. The sample size (number of independent patients) for each comparison in (g) is the same as the cluster sizes listed in (f). Test statistics and p-values for comparisons between clusters A-B, B-C, C-D, and D-E respectively are A-B: 34.1, <1e-16, B-C: 46.4, <1e-16, C-D: 10.3, <1e-16, D-E: 8.6, <1e-16, for length of stay; A-B: 262, 1e-16, B-C: 25.5, 4e-7, C-D:15.6, 7e-5, D-E: 30.0, 4e-8 for complication rates;, A-B: 7.9, 0.005, B-C: 9.8, 0.002, C-D: 31.4, 2e-8, D-E: 54.3, 1e-13 for reoperation rates; and A-B: 0.48, 0.49, B-C: 13.1, 3e-4, C-D: 1.3, 0.25, D-E: 23.1, 1e-6 for mortality rates. Error bars in (g) denote the upper bound of the 95% confidence interval on each mean. Source data are provided as a Source Data file. WBC: white blood cell count, RDW: red cell distribution width, HCT: hematocrit, PLT: platelet count, ANION: anion gap, BUN: blood-urea nitrogen, CRE: creatinine, GLU: glucose, CABG: coronary artery bypass graft.

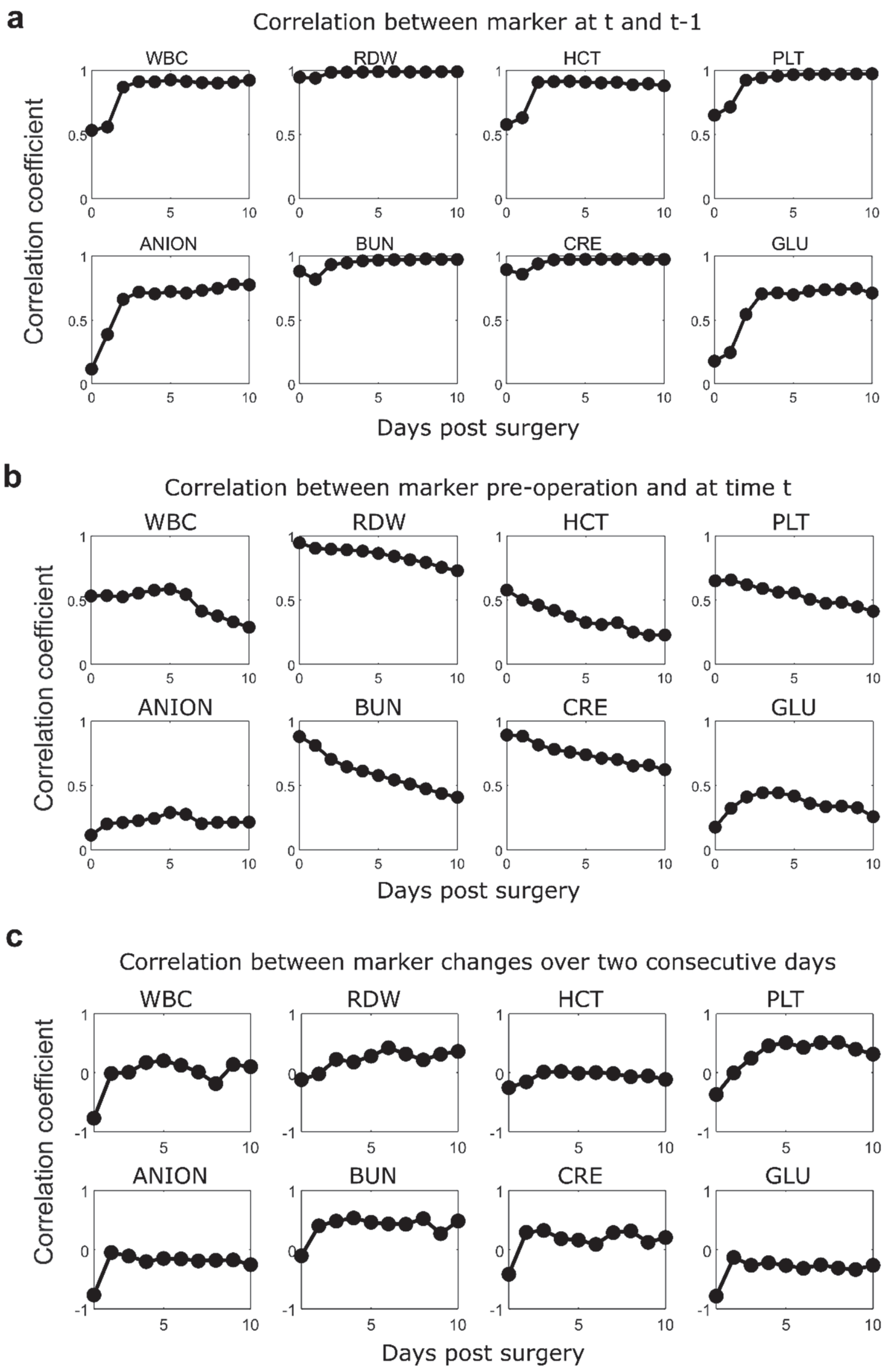

Supplementary Fig. 2 | Autocorrelations for test results throughout recovery from cardiac surgery. Correlation coefficients are given for 8 tests (WBC, RDW, HCT, PLT, ANION, BUN, CRE, GLU) throughout recovery from cardiac surgery. Correlations are provided between values over consecutive days (**a**), between current and baseline values (**b**) and between marker changes over consecutive days (**c**). All markers exhibit high autocorrelation over consecutive days, with four markers (RDW, PLT, BUN, CRE) having correlations continually above 0.9, reflecting slower dynamics than the other four markers. Three of the markers (WBC, ANION, GLU) also exhibit a type of 'memory', where correlation of day 5 values with pre-operative values is higher than correlations in the preceding days. This pattern may reflect a homeostatic memory, whereby patients return to their baseline. Four markers (RDW, PLT, BUN CRE) show high correlations between changes over consecutive days, reflecting a high momentum for these markers. Source data are provided as a Source Data file. WBC: white blood cell count, RDW: red cell distribution width, HCT: hematocrit, PLT: platelet count, ANION: anion gap, BUN: blood-urea nitrogen, CRE: creatinine, GLU: glucose.

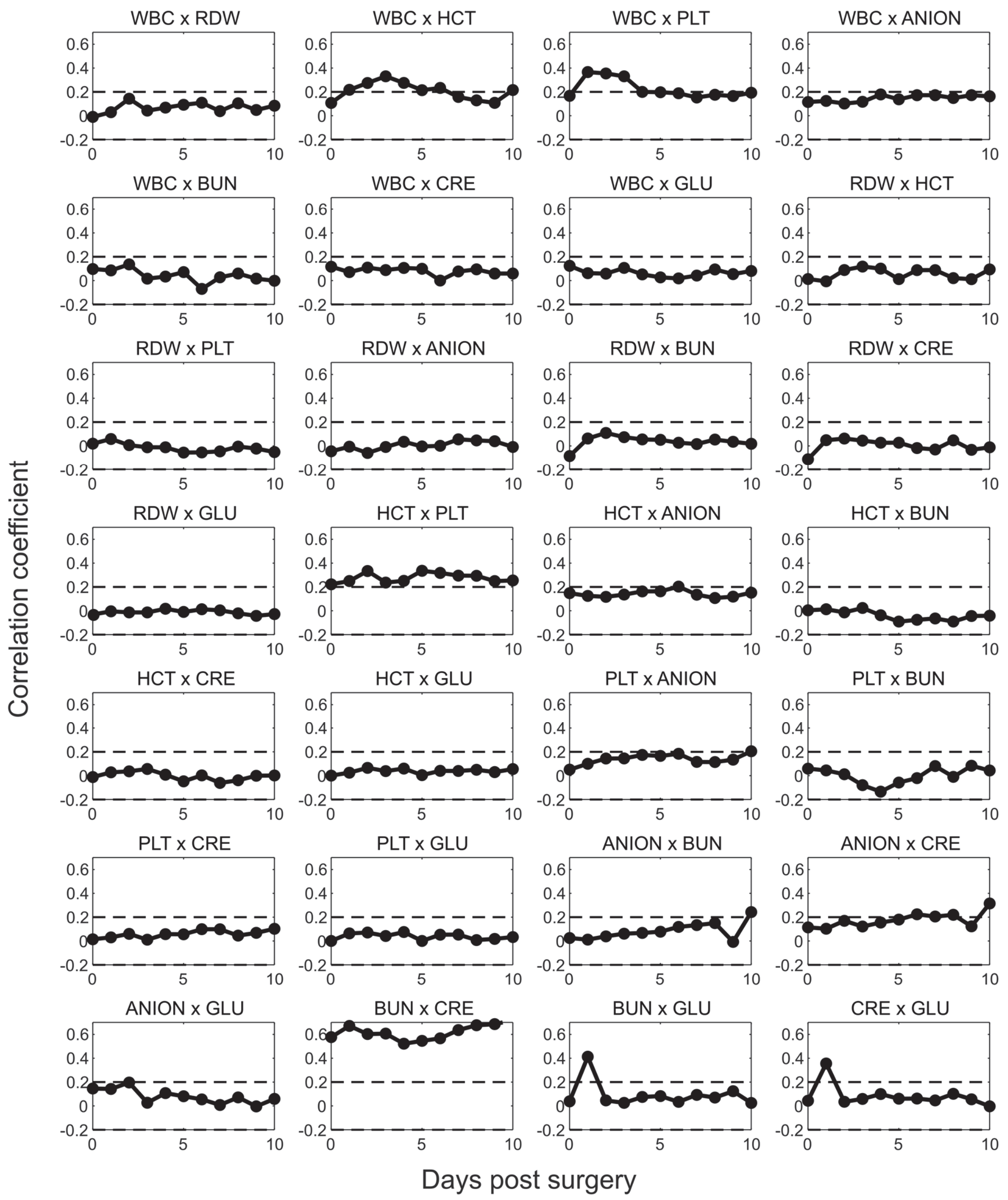

Supplementary Fig. 3 | Cross-correlations for test results throughout recovery from cardiac surgery. Cross-correlation coefficients are given for daily changes in 8 biomarkers (WBC, RDW, HCT, PLT, ANION, BUN, CRE, GLU) throughout recovery from cardiac surgery. Coefficients are the correlations between the change in each pair of results over the preceding 24hr period. Most result pairs show low cross-correlation, except for blood cell populations (WBC x HCT, WBC x PLT, HCT x PLT), suggesting strong coregulation of blood cell populations, and renal function tests (BUN x CRE). Source data are provided as a Source Data file. WBC: white blood cell count, RDW: red cell distribution width, HCT: hematocrit, PLT: platelet count, ANION: anion gap, BUN: blood-urea nitrogen, CRE: creatinine, GLU: glucose

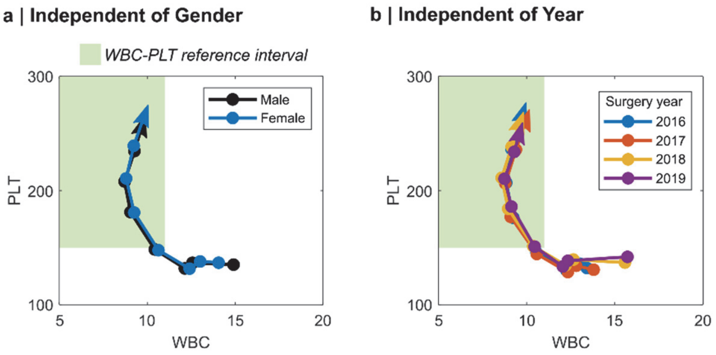

Supplementary Fig. 4 | Mean WBC-PLT trajectories for cardiac surgery patients stratified by gender, or surgery year. Similar to the results in **Fig. 1c**, the mean WBC-PLT trajectory for patients with favorable outcomes is not significantly influenced by patient gender, or the year in which the surgery took place. Source data are provided as a Source Data file. WBC: white blood cell count, PLT: platelet count.

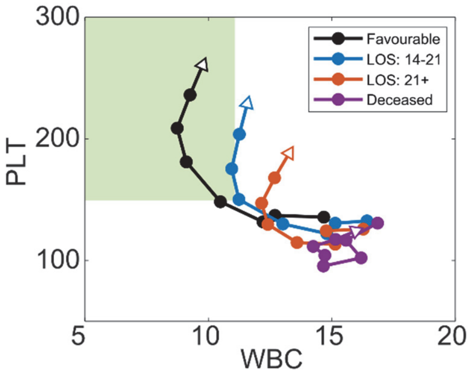

Supplementary Fig. 5 | Mean WBC-PLT trajectories for cardiac surgery patients with unfavorable outcomes. Mean trajectories are given for patients who survive with post-op hospital stays of 2-3 weeks, greater than 3 weeks, and for patients who do not survive. For comparison, the reference trajectory (**Fig. 1c**) is also included. Unlike the results in **Fig. 1c**, there is much lower coherence between the mean trajectories of patients with different unfavorable outcomes, with each set of unfavourable outcomes being distinct from the favourable outcome trajectory. Source data are provided as a Source Data file. WBC: white blood cell count, PLT: platelet count, LOS: length of post-op hospital stay.

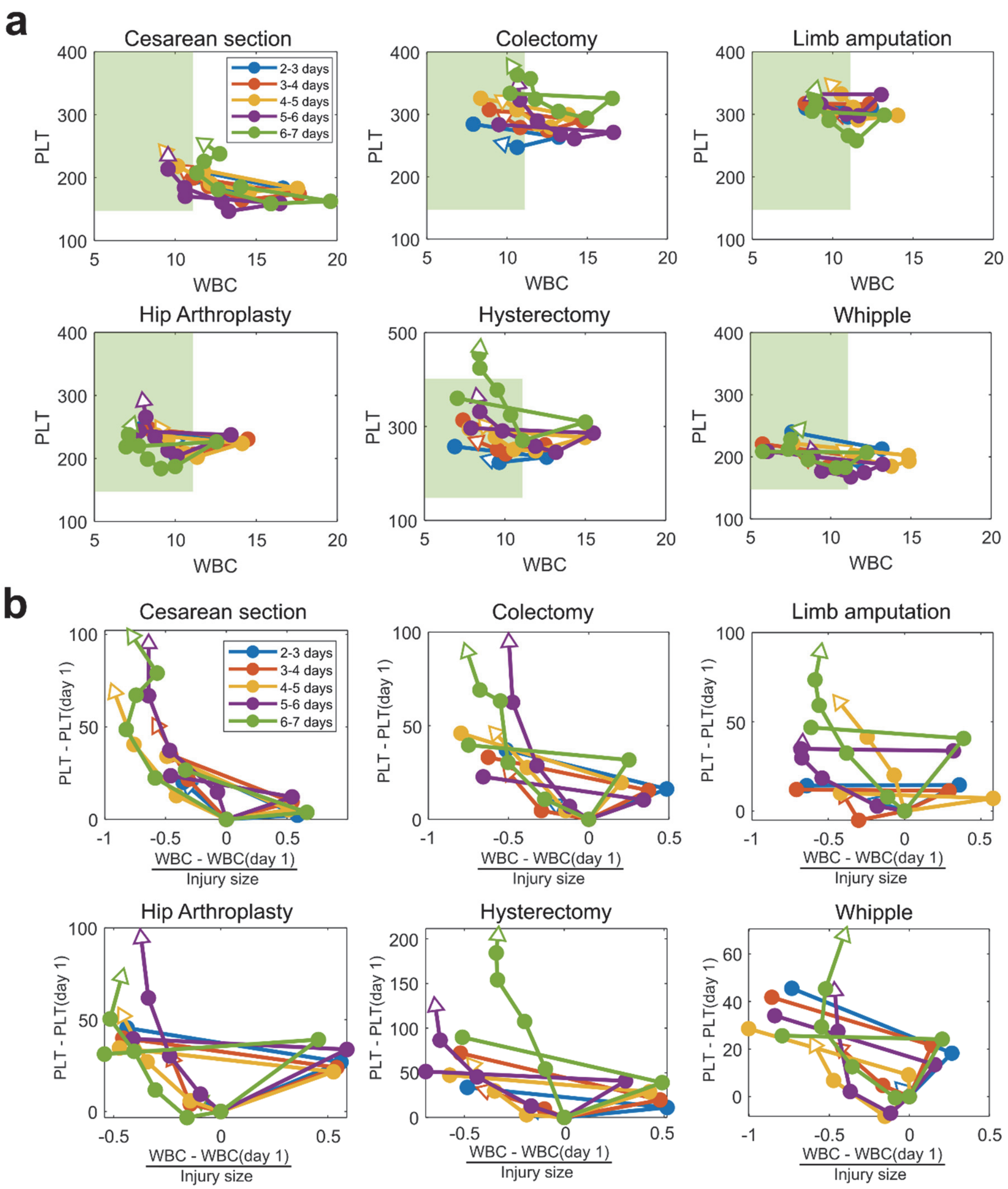

Supplementary Fig. 6 | Stratification of surgical cohort WBC-PLT trajectories by length of hospital stay (LOS). Results are presented for patients with LOS of 2-3, 3-4, 4-5, 5-6, and 6-7 days, without normalization (A), and with normalization (B) based on post-op day 1 WBC and PLT, and injury size (post-op WBC – pre-op WBC). While patients with different LOS have different baseline WBC-PLT and injury size, the shape of the mean normalized trajectories are consistent for each of the 6 surgery cohorts. Source data are provided as a Source Data file. WBC: white blood cell count, PLT: platelet count.

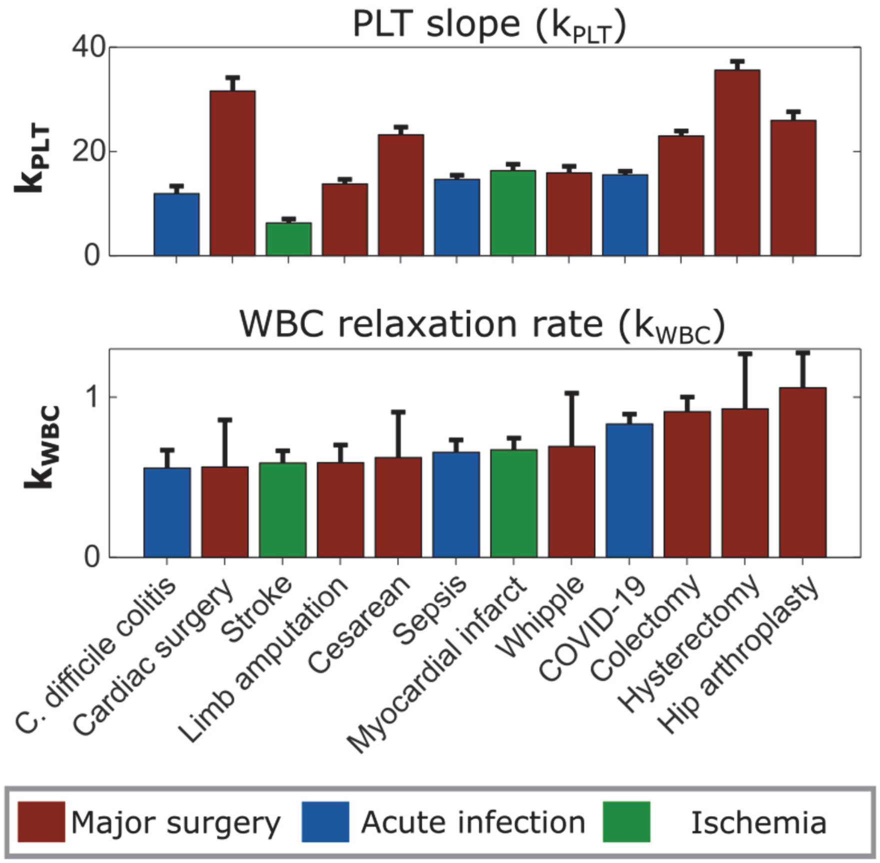

Supplementary Fig. 7 | Fitted WBC and PLT parameters for the mean recovery response in the 12 inflammatory cohorts. Model fits for k_{WBC} and k_{PLT} under an exponential decay model (WBC) and delayed linear growth model (PLT) are given, based on the mean response for each cohort. Error bars reflect the upper bound of a 95% confidence interval on each parameter. Each parameter was estimated using n = 11 data points, derived from the mean of each cohort. Cohort sizes from which the mean was generated are given in Supplementary Table 6. Corresponding illustrations of the data fits are included in **Fig. 2**, and **Fig. S12**. There are substantial differences in PLT slope across the 12 cohorts, but the differences in WBC relaxation rate are less substantial. Source data are provided as a Source Data file. WBC: white blood cell count, PLT: platelet count.

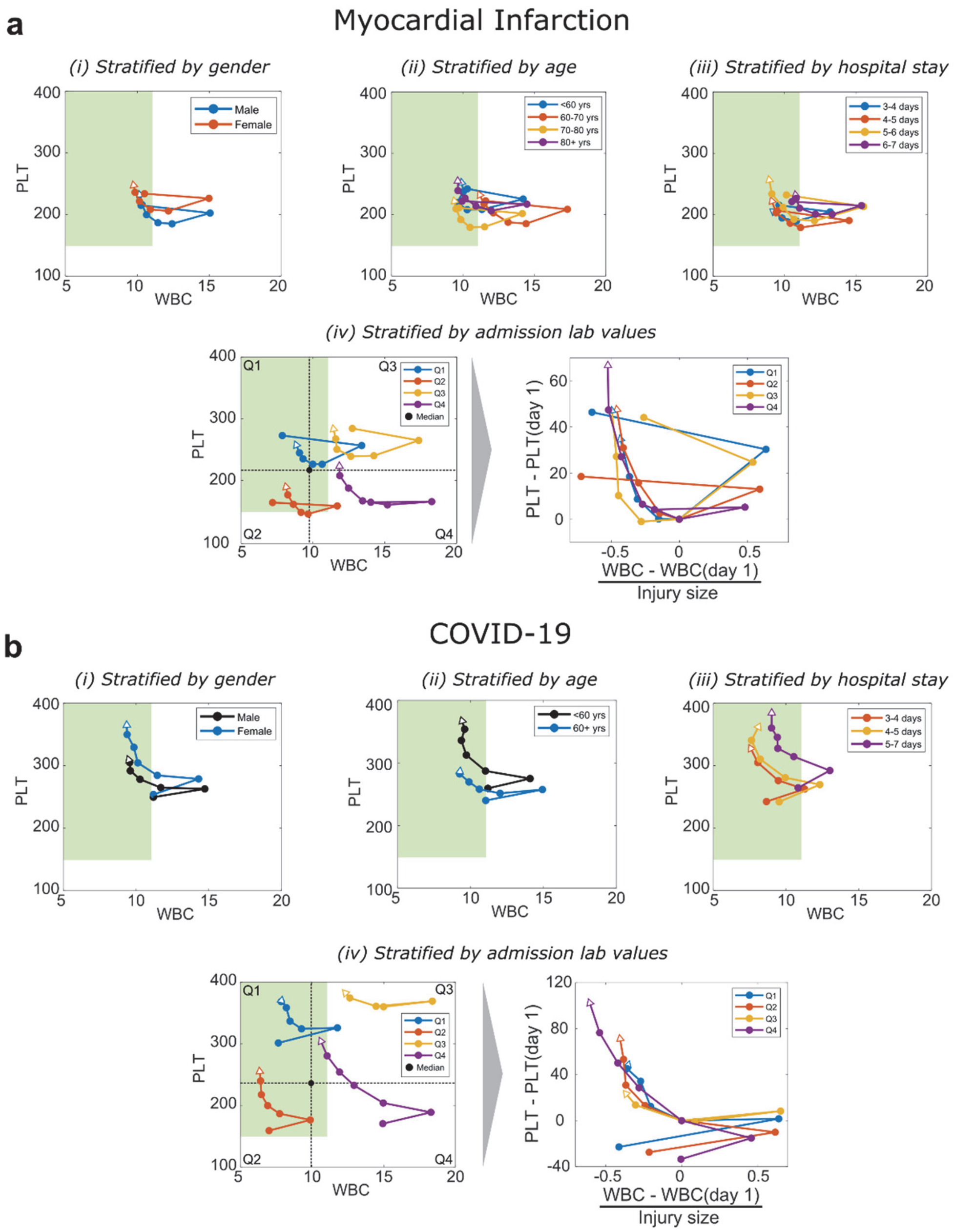

Supplementary Fig. 8 | Mean WBC-PLT trajectories stratified by demographic and clinical factors for myocardial infarction and COVID-19 cohorts. Stratification by gender, age, and LOS is associated with admission WBC-PLT, but the shape of the mean trajectory is similar independent of gender, age, LOS, and admission WBC-PLT. Both myocardial infarction (**a**) and COVID-19 (**b**) cohorts have been aligned as described in the main text methods. Source data are provided as a Source Data file. WBC: white blood cell count, PLT: platelet count.

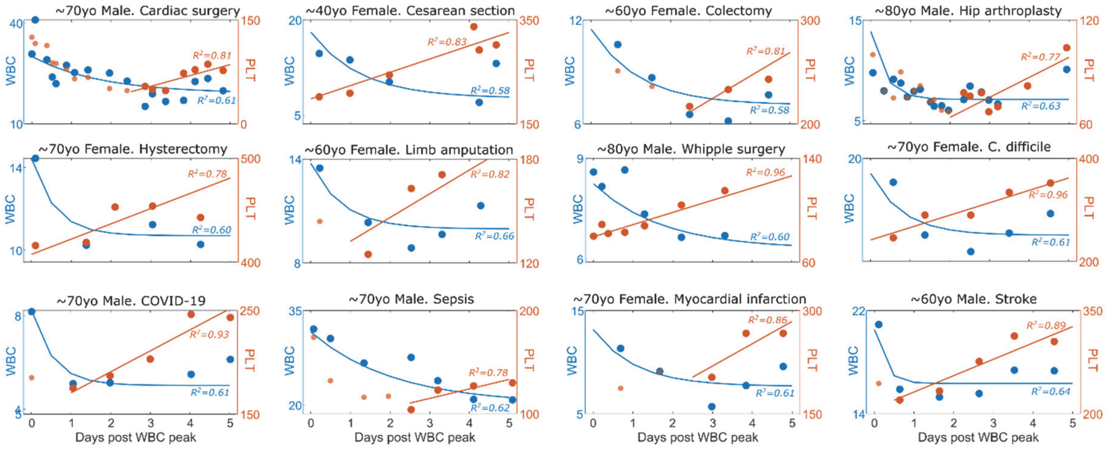

Supplementary Fig. 9 | Example patient trajectories and model fits for patients whose raw data had goodness of fit with adjusted R^2 near the 25th percentile (poorest fitting quartile). Raw patient data and corresponding WBC and PLT model fits are given for a patient in each cohort whose model fit had an adjusted R^2 closest to the 25th percentile for PLT R^2 (0.6) and WBC R^2 (0.82). Despite having poorer fits than ~75% of the cohort, the pattern of WBC exponential decay and delayed linear PLT increase is apparent. WBC: white blood cell count, PLT: platelet count.

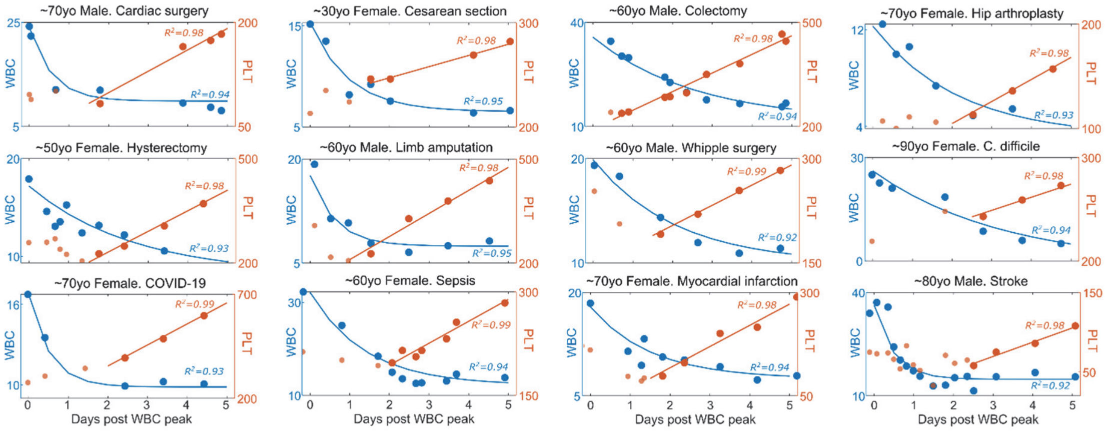

Supplementary Fig. 10 | Example patient trajectories and model fits for patients whose raw data had goodness of fit with adjusted R^2 near the 75th percentile (best fitting quartile). Raw patient data and corresponding WBC and PLT model fits are given for a patient in each cohort whose model fit had an adjusted R^2 closest to the 75th percentile for PLT R^2 (0.98) and WBC R^2 (0.94). WBC: white blood cell count, PLT: platelet count.

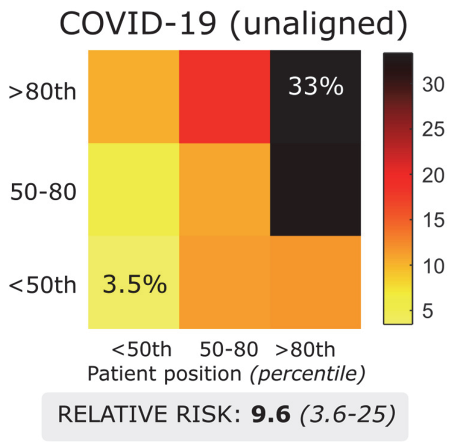

Supplementary Fig. 11 | COVID-19 mortality risk stratification by position and direction percentiles without alignment. For completeness, risk stratification of the COVID-19 cohort was calculated without aligning the cohort based on their peak WBC values (within 72hrs of admission), as was done in **Fig. 3**. While the exact prevalence rates differ from the aligned results in **Fig 3**, the overall magnitude of risk stratification from patients with position and direction < 50th relative to those with position and direction > 80th is similar. Source data are provided as a Source Data file.

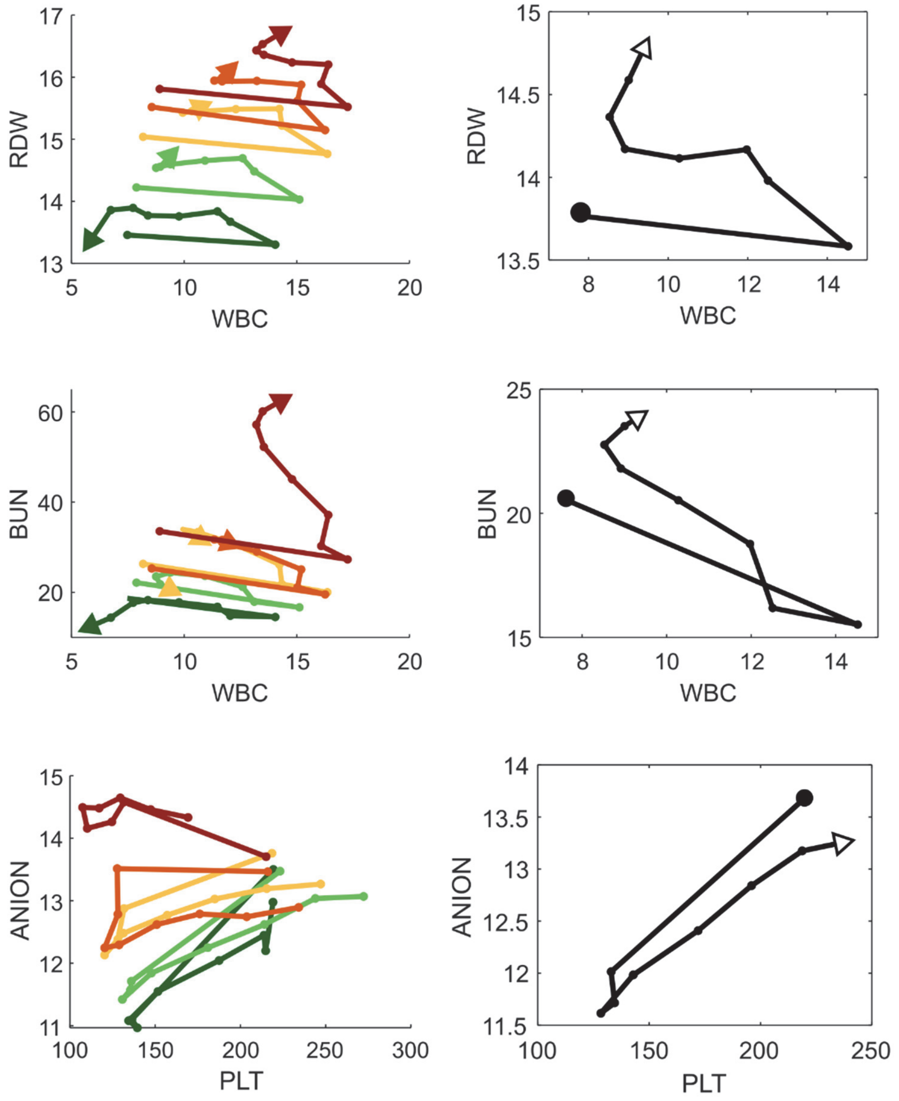

Supplementary Fig. 12 | Cardiac surgery recovery trajectories for alternate test result pairs. Mean trajectories for each cluster (left) and for patients with good outcomes (right) are given for three alternate combinations of test results: WBC x RDW, WBC x BUN, and PLT x ANION. Each trajectory is from preop to day 7, with spacing between dots equal to 1 day. Source data are provided as a Source Data file. WBC: white blood cell count, PLT: platelet count.

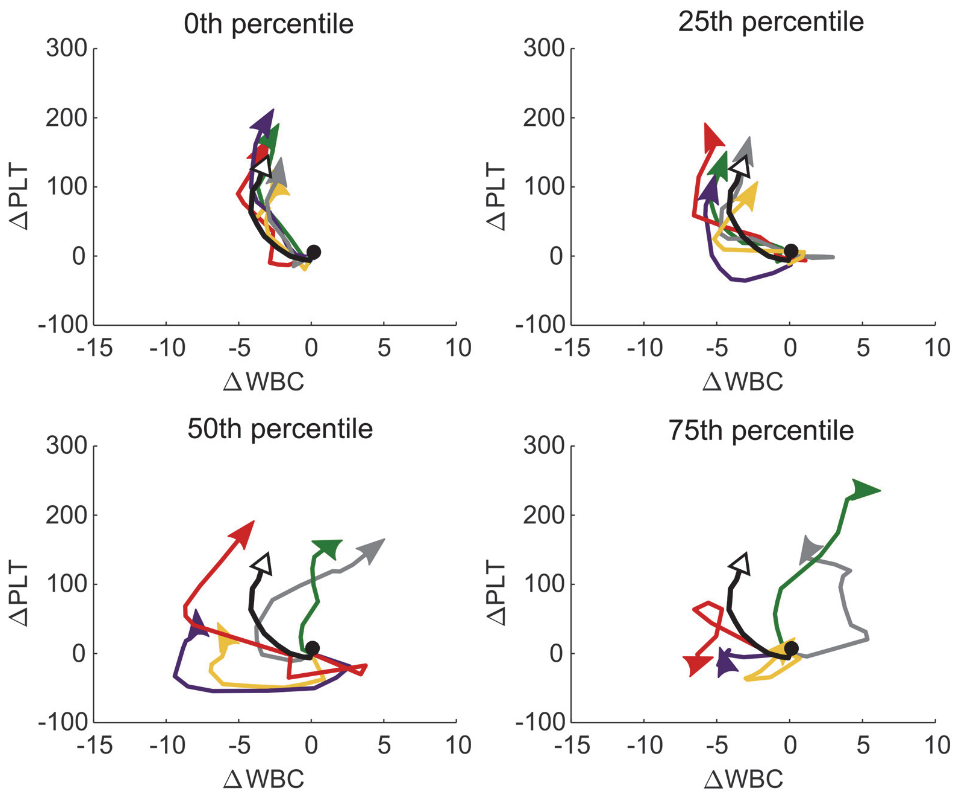

Supplementary Fig. 13 | Example cardiac surgery WBC-PLT trajectories stratified by average degree of deviation for the full cohort. The 5 patient trajectories closest to the 0th, 25th, 50th and 75th percentile of average deviation (from day 1 to day 7) from the mean WBC-PLT trajectory are given, from post op day 1 to day 7. The 0th and 25th percentile trajectories adhere closely to the mean trajectory. The 50th percentile exhibits high variance early on, but eventually adheres to the shape of the mean trajectory. No consistent patterns are seen in the 75th percentile trajectory. Patient selection was limited to MGH cohort patients with hospital stays > 7 days, and as such is biased towards adverse trajectories. WBC: white blood cell count, PLT: platelet count.

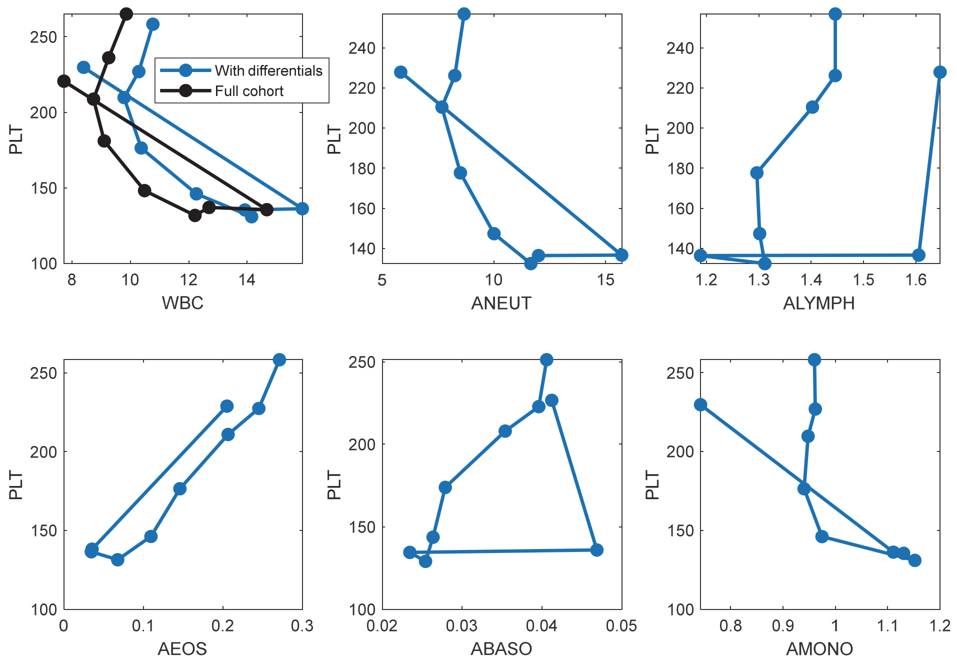

Supplementary Fig. 14 | Phase-plane trajectories for cardiac surgery patients with favourable outcomes using WBC sub-types from WBC differentials. Trajectories are given for absolute counts of neutrophils (ANEUT), lymphocytes (ALYMPH), eosinophils (AEOS), basophils (ABASO), and monocytes (AMONO) versus PLT. WBC differentials were not available at the same frequency as standard blood counts, and analysis was limited to patients with differentials available preop and until at least day 7 post-op (N = 225). For reference the WBC-PLT trajectory of this cohort is compared to the overall cohort and shows the same shape. Neutrophils contribute most strongly to the overall WBC count and follow the same trajectory shape. This same shape is followed broadly by monocytes as well. Source data are provided as a Source Data file. WBC: white blood cell count, PLT: platelet count.

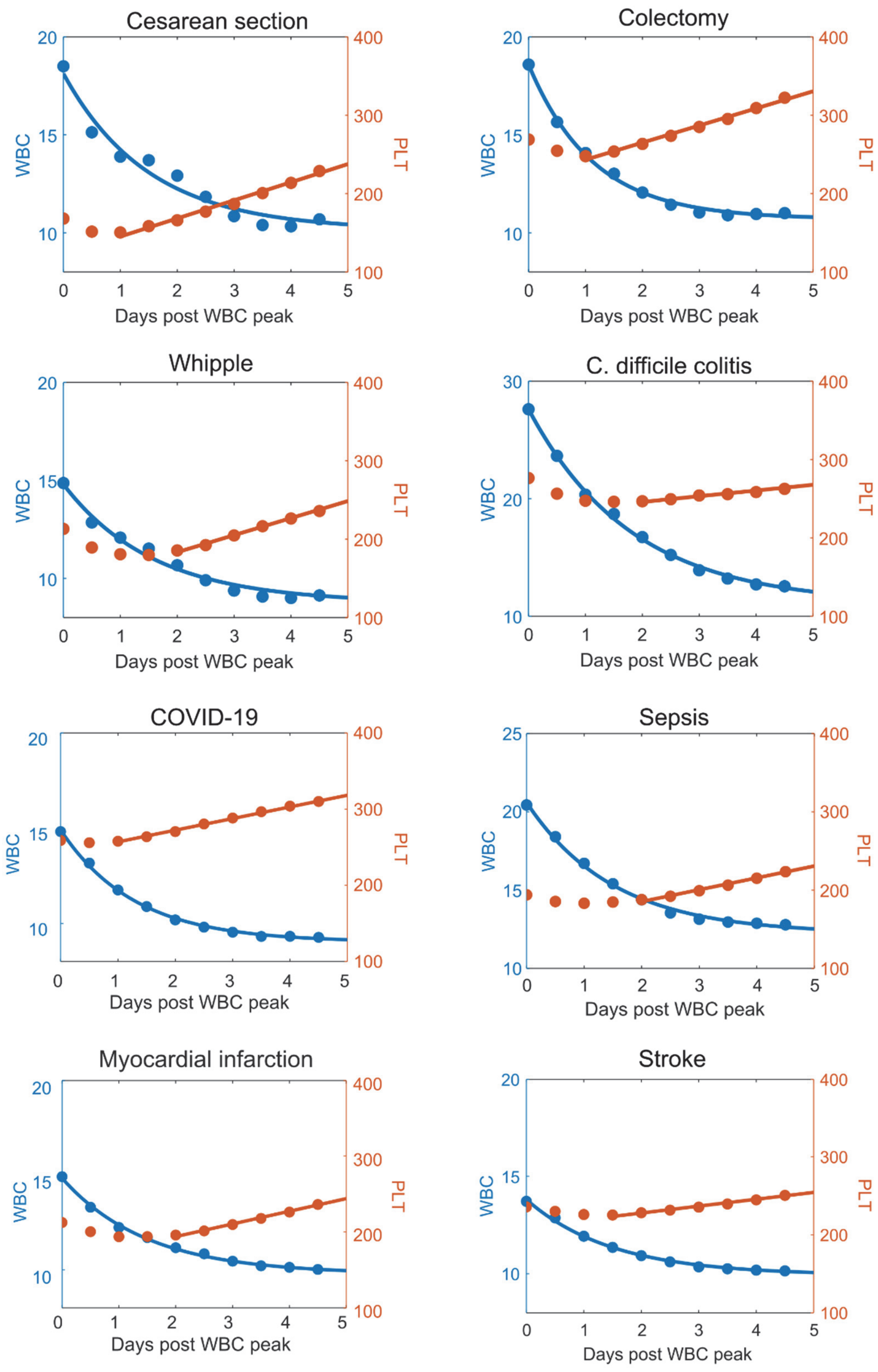

Supplementary Fig. 15 | Exponential and linear model fits for other inflammatory cohorts. Mean WBC and PLT data for 5 days post WBC peak are given for 8 inflammatory cohorts: Cesarean, colectomy, Whipple procedure, stroke, C. difficile colitis, COVID-19, sepsis, myocardial infarction, and stroke. Fits of an exponential model (WBC) and linear model (PLT) are included. Corresponding fit parameters are included in **Fig. S7**. Source data are provided as a Source Data file. WBC: white blood cell count, PLT: platelet count.

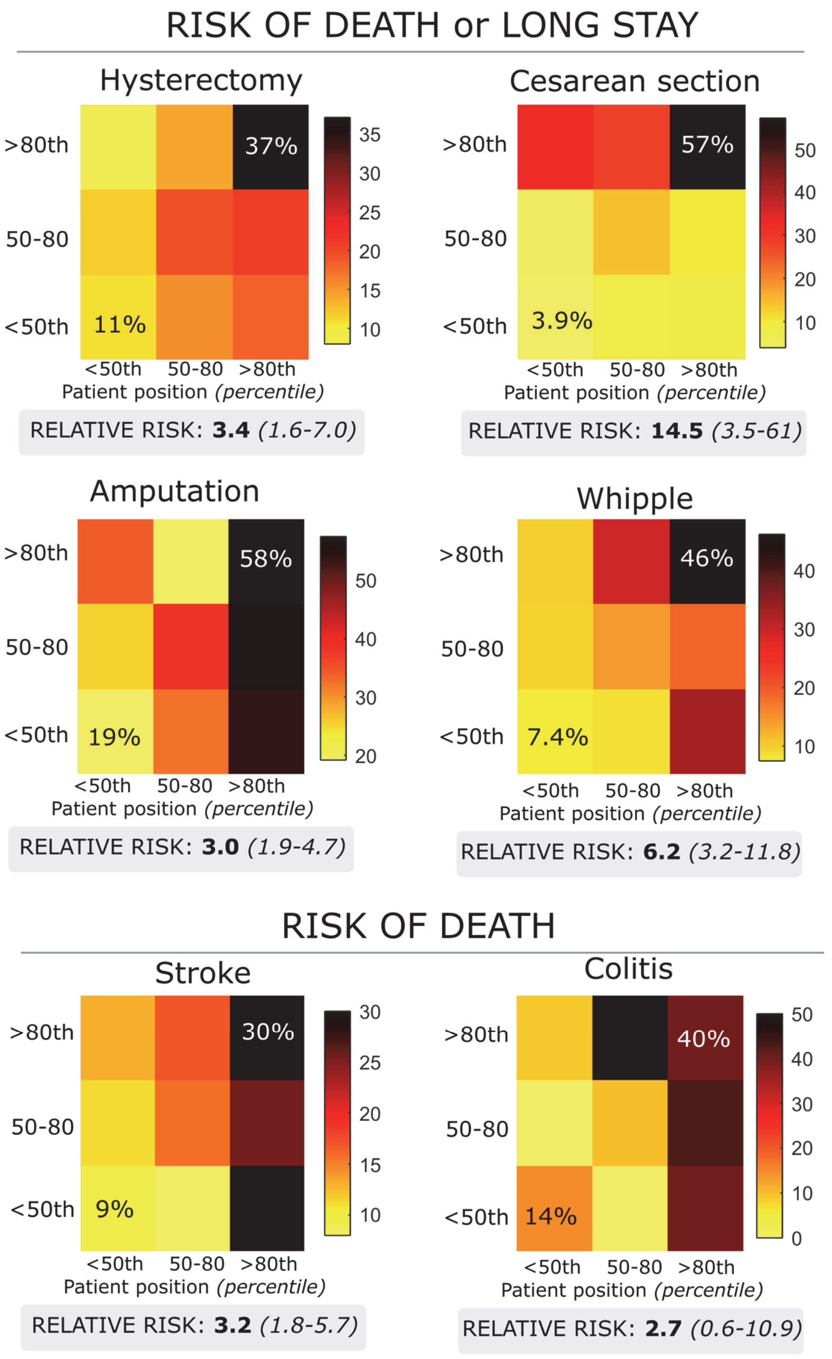

Supplementary Fig. 16 | Risk of adverse outcomes stratified by day 4 positional and directional risk. In similar format to **Fig 3**, patient risk of death or long stay, stratified by day 4 positional and directional percentiles are given for the remaining 6 inflammation cohorts. In each case, patients with position and direction above the 80th percentile have significantly elevated risk comparative to patients whose position and direction is below the 50th percentile. Note that due to sample size constraints, only stroke results are out-of-sample (where thresholds were defined in the exploratory cohort, and outcomes calculated from the validation cohort). For the other cohorts, thresholds and outcome rates were calculated from the overall cohort – and further validation in a larger scale study is needed. Source data are provided as a Source Data file.

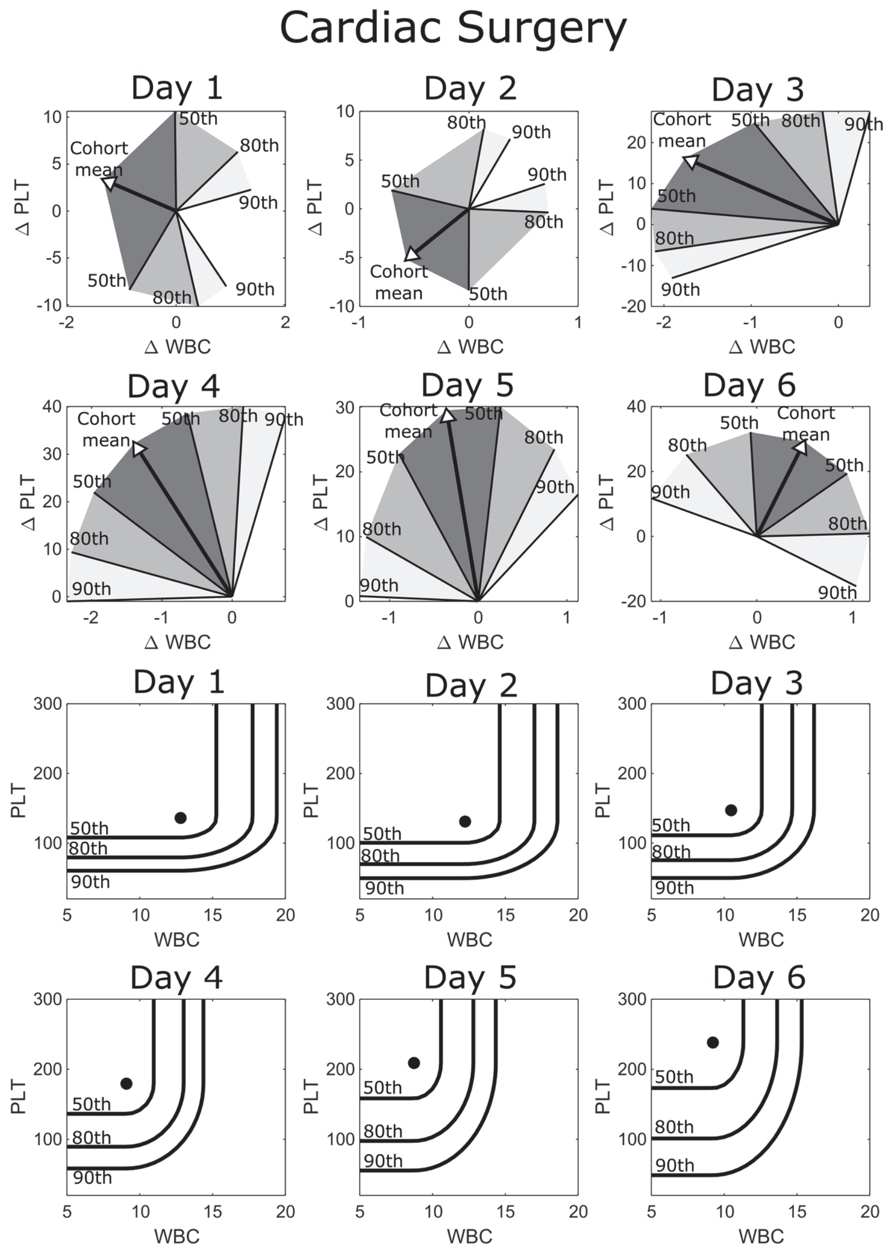

Supplementary Fig. 17 | Reference position and direction percentiles charts for post-op days 1 through 6 for cardiac surgery. Source data are provided as a Source Data file. WBC: white blood cell count, PLT: platelet count.

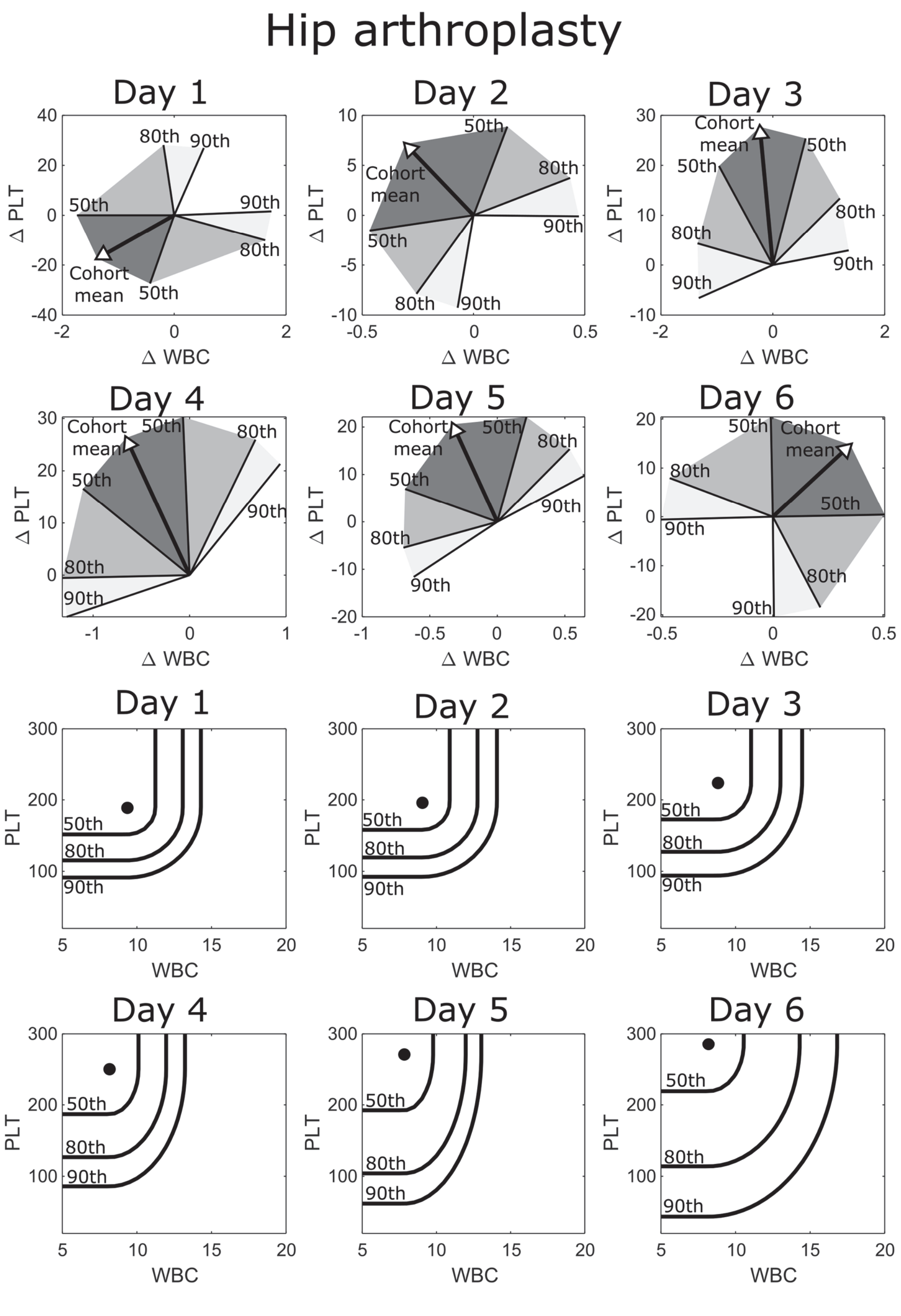

Supplementary Fig. 18 | Reference position and direction percentiles charts for post-op days 1 through 6 for hip arthroplasty. Source data are provided as a Source Data file. WBC: white blood cell count, PLT: platelet count.

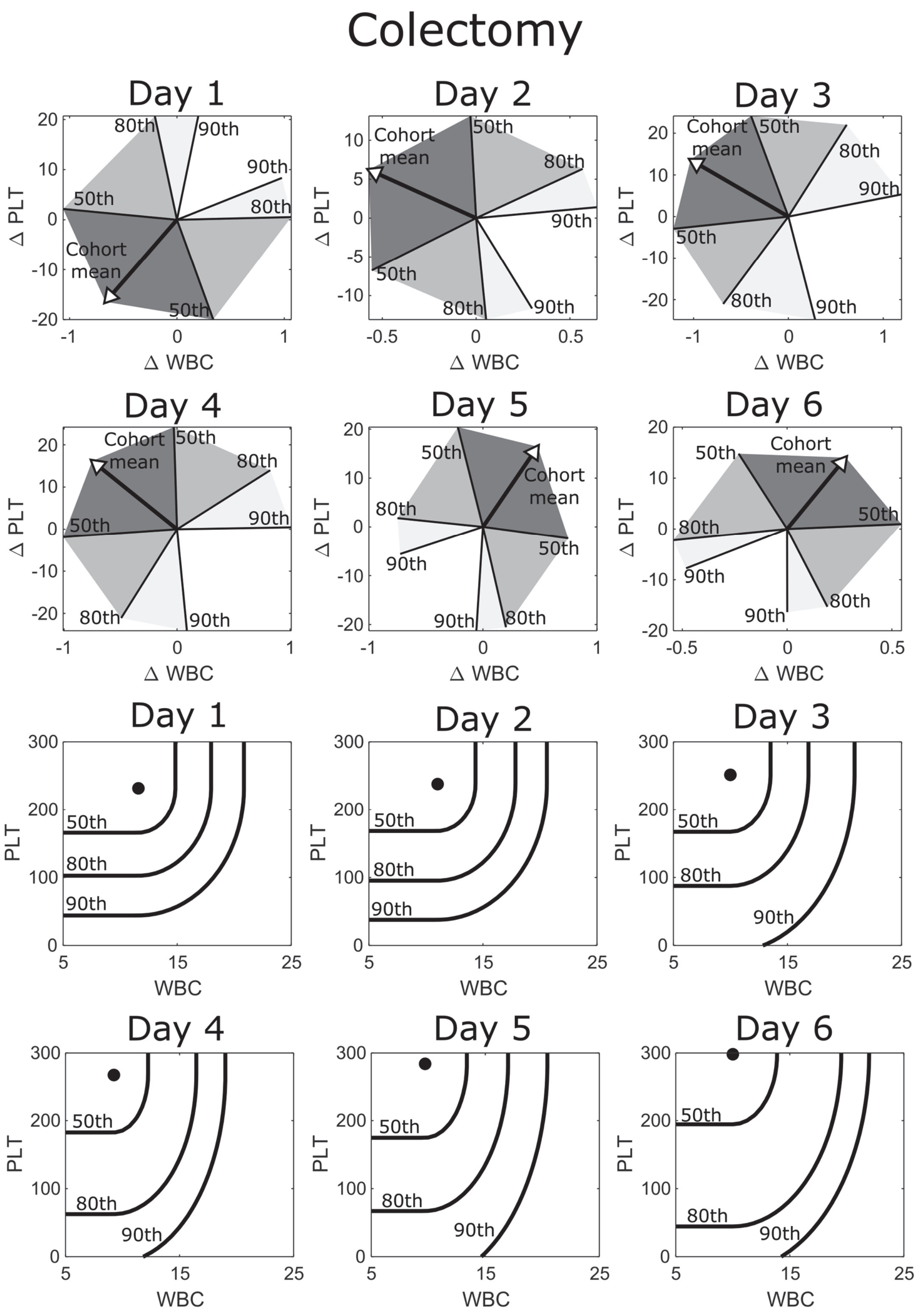

Supplementary Fig. 19 | Reference position and direction percentiles charts for post-op days 1 through 6 for colectomy. Source data are provided as a Source Data file. WBC: white blood cell count, PLT: platelet count.

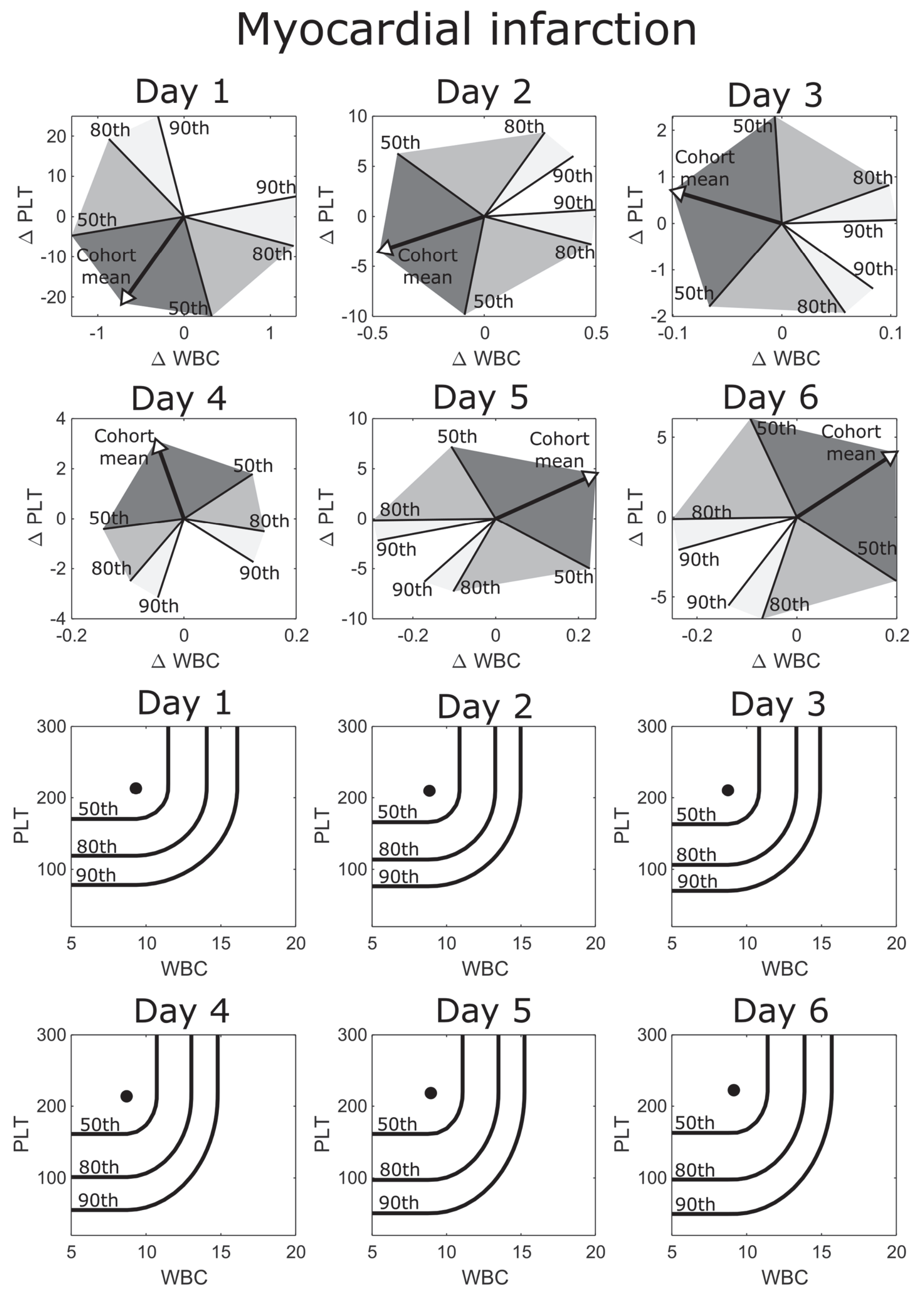

Supplementary Fig. 20 | Reference position and direction percentiles charts for post admission days 1 through 6 for myocardial infarction. Source data are provided as a Source Data file. WBC: white blood cell count, PLT: platelet count.

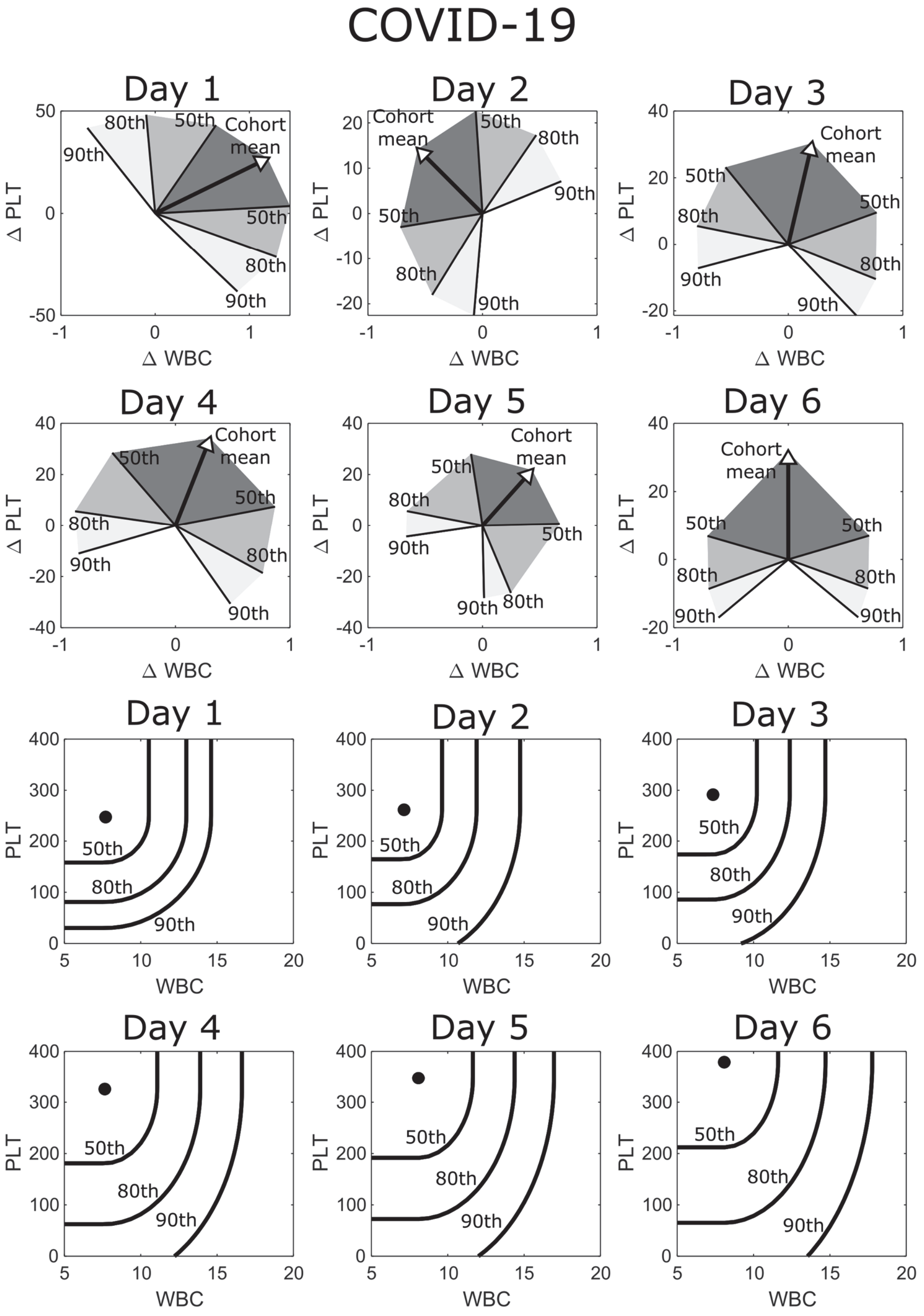

Supplementary Fig. 21 | Reference position and direction percentiles charts for post admission days 1 through 6 for COVID-19, post alignment using maximum WBC count during first 72hrs. Source data are provided as a Source Data file. WBC: white blood cell count, PLT: platelet count.

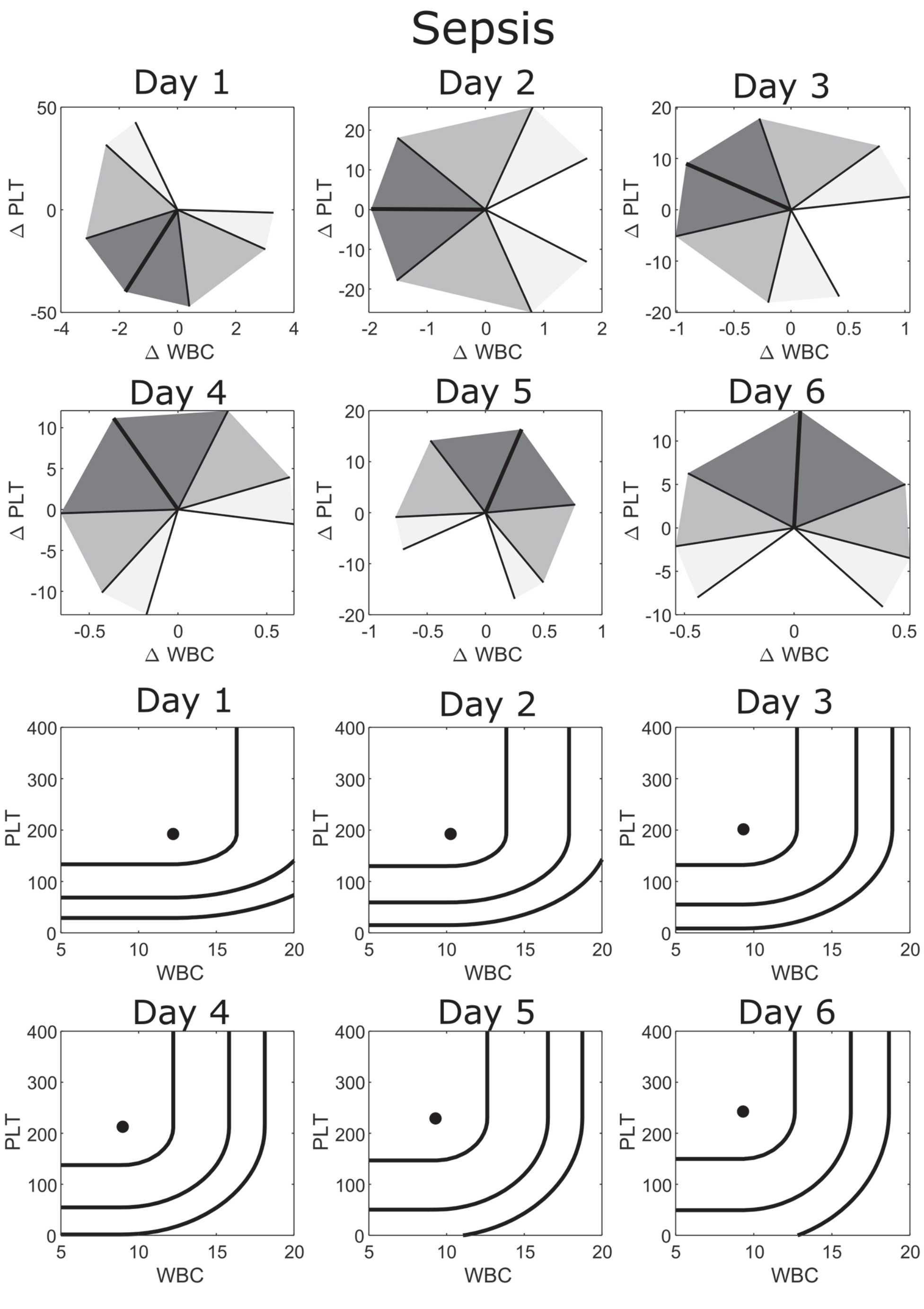

Supplementary Fig. 22 | Reference position and direction percentiles charts for post admission days 1 through 6 for sepsis, post alignment using maximum WBC count during first 72hrs. Source data are provided as a Source Data file. WBC: white blood cell count, PLT: platelet count.

Supplementary Tables

Supplementary Table 1 | Cardiac surgery high-dimensional cluster characteristics

**Basic Characteristics**	Total	Cluster A	Cluster B	Cluster C	Cluster D	Cluster E
No. of patients (%)	4693 (100%)	2393 (51%)	1217 (26%)	528 (11%)	347 (7.5%)	208 (4.5%)
Age - mean (SD) yrs	63.9 (13)	62.3 (12.7)	66.4 (13)	65.7 (12.7)	62.1 (14.4)	66.4 (12)
Gender - No. Male (%)	3344 (71.3)	1778 (74.3)	854 (70.2)	360 (68.2)	209 (60.2)	143 (68.8)
Race - No. White/Caucasian (%)	4047 (86.2)	2093 (87.5)	1063 (87.3)	442 (83.7)	275 (79.3)	174 (83.7)
Major category surgeries - No. (%)	2959 (63.1)	1732 (72.4)	780 (64.1)	250 (47.3)	109 (31.4)	88 (42.3)
**Pre-operative risk factors**						
Smoking history - No. (%)	2508 (53.4)	1175 (49.1)	715 (58.8)	306 (58)	188 (54.2)	124 (59.6)
Diabetic - No. (%)	1359 (29)	555 (23.2)	406 (33.4)	174 (33)	120 (34.6)	104 (50)
Lung disease - No. (%)	680 (14.5)	209 (8.7)	196 (16.1)	109 (20.6)	98 (28.2)	68 (32.7)
Dyslipidemia - No. (%)	3589 (76.5)	1776 (74.2)	968 (79.5)	417 (79)	255 (73.5)	173 (83.2)
Endocarditis - No. (%)	267 (5.7)	71 (3)	82 (6.7)	47 (8.9)	40 (11.5)	27 (13)
Family history of coronary artery disease - No. (%)	562 (12)	339 (14.2)	126 (10.4)	53 (10)	27 (7.8)	17 (8.2)
Hypertension - No. (%)	3643 (77.6)	1779 (74.3)	992 (81.5)	432 (81.8)	258 (74.4)	182 (87.5)
Pulmonary vascular disease - No. (%)	560 (11.9)	188 (7.9)	179 (14.7)	87 (16.5)	56 (16.1)	50 (24)
Renal Failure - No. (%)	89 (1.9)	3 (0.1)	22 (1.8)	28 (5.3)	19 (5.5)	17 (8.2)
**Pre-operative biomarkers**						
White blood cell count- mean (SD) $10^3/\mu L$	7.9 (3.1)	7.5 (2.2)	8 (3.1)	8.2 (2.9)	8.7 (5.3)	9 (3.4)
Red cell distribution width - mean (SD) %	14.2 (2.2)	13.5 (1.3)	14.3 (2)	15.1 (3)	15.6 (2.5)	15.8 (3)
Hematocrit - mean (SD) %	38.1 (6.1)	40.5 (4.8)	37.4 (5.9)	36.2 (6.3)	34.3 (6.7)	33.5 (6.4)
Platelet count - mean (SD) $10^3/\mu L$	217.9 (71.7)	216.7 (61.9)	221.9 (77.5)	216.6 (73.6)	216.5 (85.4)	214.5 (86.2)
Anion gap - mean (SD) mmol/L	13.5 (2.5)	13.4 (2.5)	13.5 (2.4)	13.8 (2.7)	13.5 (2.4)	13.8 (2.6)
Blood-urea nitrogen - mg/dL	22.4 (12.1)	18.6 (7.2)	22 (10.7)	26.2 (14.9)	25.2 (14.3)	33.7 (18.2)
Creatinine - mean (SD) mg/dL	1.2 (0.9)	1 (0.3)	1.2 (0.8)	1.5 (1.3)	1.5 (1.3)	1.7 (1)
Glucose - mean (SD) mg/dL	126.2 (41.9)	122.7 (39.3)	127 (41.9)	129.6 (44.8)	127.1 (46.9)	133.3 (41.6)
**Post-operative outcomes**						
Inpatient stay - mean (SD) days	8.8 (10.4)	4.9 (2.3)	7.4 (1.2)	11 (2.1)	20.5 (20.9)	36.4 (21.4)
30-day mortality - No. (%)	107 (2.3)	20 (0.8)	13 (1.1)	19 (3.6)	18 (5.2)	37 (17.8)
Renal failure - No. (%)	140 (3)	13 (0.5)	9 (0.7)	24 (4.5)	14 (4)	80 (38.5)
Permanent stroke - No. (%)	87 (1.9)	10 (0.4)	16 (1.3)	20 (3.8)	22 (6.3)	19 (9.1)
>24hr ventilation - No. (%)	458 (9.8)	32 (1.3)	59 (4.8)	89 (16.9)	122 (35.2)	156 (75)
Reoperation - No. (%)	293 (6.2)	46 (1.9)	42 (3.5)	36 (6.8)	67 (19.3)	102 (49)

Legend: SD: standard deviation.

Supplementary Table 2 | Adverse outcome stratification by day 4 position and direction percentiles for various test result combinations in the cardiac surgery cohort

		Adverse outcome likelihood		
Marker 1	Marker 2	Position and direction < 50th percentile	Position and direction > 80th percentile	Relative risk
PLT	WBC	1.8%	51.6%	29.4
RDW	WBC	1.8%	53.8%	29.3
WBC	BUN	2.3%	49.4%	21.5
PLT	ANION	3.8%	69.0%	18.3
PLT	BUN	3.1%	55.5%	18.2
WBC	GLU	2.1%	33.7%	16.3
WBC	ANION	3.3%	52.0%	15.9
PLT	GLU	3.5%	50.4%	14.3
HCT	WBC	3.5%	48.3%	13.6
WBC	CRE	3.6%	47.3%	13.3
PLT	RDW	3.5%	43.7%	12.4
HCT	PLT	4.0%	48.7%	12.1
HCT	RDW	2.2%	26.7%	12.1
ANION	GLU	5.5%	47.4%	8.7
PLT	CRE	5.3%	46.3%	8.7
RDW	GLU	4.2%	25.0%	5.9
ANION	CRE	9.0%	50.0%	5.5
HCT	ANION	5.3%	27.3%	5.2
ANION	BUN	4.8%	21.7%	4.5
RDW	ANION	5.1%	17.6%	3.5
BUN	GLU	4.9%	16.7%	3.4
HCT	GLU	8.1%	25.8%	3.2
BUN	CRE	6.6%	18.8%	2.8
RDW	CRE	7.4%	20.0%	2.7
CRE	GLU	10.8%	24.8%	2.3
HCT	BUN	3.7%	7.4%	2.0
RDW	BUN	2.3%	4.5%	1.9
HCT	CRE	10.4%	7.7%	0.7

Legend: WBC: white blood cell count, RDW: red cell distribution width, HCT: hematocrit, PLT: platelet count, ANION: anion gap, BUN: blood-urea nitrogen, CRE: creatinine, GLU: glucose.

Supplementary Table 3 | Outcomes stratified by day 4 position and direction percentiles for alternate 2D test result combinations in the cardiac surgery cohort

WBC x PLT

		Adverse outcomes likelihood Distance percentile			Remaining length of stay Distance percentile			Number of patients Distance percentile		
		<50th	50-80th	>80th	<50th	50-80th	>80th	<50th	50-80th	>80th
Position percentile	<50th	1.75%	3.3%	14.7%	2.1	3.0	5.9	399	211	68
	50-80th	6.8%	7.4%	30.6%	3.5	5.2	10.7	162	122	72
	>80th	25.0%	23.0%	52.6%	7.2	9.2	15.1	56	61	133

WBC x RDW

		Adverse outcomes likelihood Distance percentile			Remaining length of stay Distance percentile			Number of patients Distance percentile		
		<50th	50-80th	>80th	<50th	50-80th	>80th	<50th	50-80th	>80th
Position percentile	<50th	1.8%	1.3%	5.5%	2.3	2.3	4.1	327	223	109
	50-80th	9.7%	17.2%	24.5%	5.8	6.0	8.3	145	128	94
	>80th	27.7%	23.5%	53.8%	8.8	8.7	15.0	101	68	80

WBC x BUN

		Adverse outcomes likelihood Distance percentile			Remaining length of stay Distance percentile			Number of patients Distance percentile		
		<50th	50-80th	>80th	<50th	50-80th	>80th	<50th	50-80th	>80th
Position percentile	<50th	2.3%	2.0%	6.0%	2.3	3.1	3.3	348	204	117
	50-80th	8.3%	14.3%	29.6%	4.8	4.7	9.4	157	91	81
	>80th	21.9%	31.7%	49.4%	8.4	9.8	15.5	105	82	85

PLT x ANION

		Adverse outcomes likelihood Distance percentile			Remaining length of stay Distance percentile			Number of patients Distance percentile		
		<50th	50-80th	>80th	<50th	50-80th	>80th	<50th	50-80th	>80th
Position percentile	<50th	3.8%	8.6%	10.5%	2.8	4.0	5.9	531	175	57
	50-80th	12.2%	11.0%	21.4%	4.9	5.8	8.3	164	100	56
	>80th	15.7%	28.4%	69.0%	6.5	10.9	19.0	51	67	71

Legend: WBC: white blood cell count, RDW: red cell distribution width, PLT: platelet count, ANION: anion gap, BUN: blood-urea nitrogen.

Supplementary Table 4 | Comparison of risk stratification using WBC-PLT trajectories to PLT/WBC ratio and WBC or PLT in isolation.

Marker	Time period	Lower risk	Higher Risk	Risk ratio (95% CI)
2D WBC-PLT Trajectory	Day 0-1	3.8%	38.1%	9.9 (5.9 - 16.6)
	Day 1-2	3.5%	31.4%	8.9 (4.7 - 16.8)
	Day 2-3	1.4%	43.6%	32.2 (13.1 - 78.7)
	Day 3-4	1.6%	52.3%	33.0 (14.7 - 74.6)
PLT/WBC Ratio	Day 1	5.3%	31.7%	6.0 (3.8 - 9.4)
	Day 2	5.0%	33.3%	6.7 (4.3 - 10.4)
	Day 3	4.2%	41.5%	9.8 (6.2 - 15.5)
	Day 4	2.5%	52.9%	21.3 (11.6 - 39.0)
WBC	Day 1	9.0%	32.3%	3.6 (5.2 - 2.5)
	Day 2	8.4%	28.8%	3.4 (2.3 - 5.0)
	Day 3	6.5%	32.4%	4.9 (3.3 - 7.5)
	Day 4	5.8%	38.9%	6.7 (4.3 - 10.5)
PLT	Day 1	7.6%	26.1%	3.4 (2.2 - 5.2)
	Day 2	6.8%	29.0%	4.3 (2.8 - 6.5)
	Day 3	5.3%	34.6%	6.5 (4.2 - 10.1)
	Day 4	6.1%	44.9%	7.3 (4.8 - 11.2)

Legend: Risk ratios for the 2D WBC-PLT trajectory were calculated using the same method as in ***Fig. 4****. Ratios for the other three markers were calculated by comparing biomarker values on the given day for patients below the 30th percentile to those above the 90th percentile using the same out-of-sample testing procedure as in* ***Fig. 4.*** *30th and 90th percentiles were chosen to reflect approximately similar cohort sizes as patients with position and direction below 50th percentile or above 80th percentile in* ***Fig. 4****. WBC: white blood cell count, PLT: platelet count.*

Supplementary Table 5 | Measurement units and reference intervals for study test results, at Massachusetts General Hospital and Brigham and Women's Hospital.

			Reference interval			
			MGH		BWH	
	Abbreviation	Units	Male	Female	Male	Female
**Complete blood count**						
Hematocrit	HCT	%	41-53	36-46	40-54	36-48
Hemoglobin	HGB	g/dL	13.5-17.5	12.0-16.0	13.5-18	11.5-16.4
Mean corpuscular hemoglobin	MCH	pg	26-34	26-34	27-32	27-32
Mean corpuscular hemoglobin concentration	MCHC	g/dL	31-37	31-37	32-36	32-36
Mean corpuscular volume	MCV	fL	80-100	80-100	80-95	80-95
Mean platelet volume	MPV	fL	8.4-12.0	8.4-12.0	8.4-12.0	8.4-12.0
Platelet count	PLT	10^3/µL	150-400	150-400	150-400	150-400
Red blood cell count	RBC	10^6/µL	4.5-5.9	4.0-5.2	3.9-6.0	4.5-6.4
Red cell distribution width	RDW	%	11.5-14.5	11.5-14.5	11.5-14.5	11.5-14.5
White blood cell count	WBC	10^3/µL	4.5-11.0	4.5-11.0	4.0-10.0	4.0-10.0
**Basic metabolic panel**						
Anion gap	ANION	mmol/L	3-17	3-17	3-17	3-17
Blood-urea nitrogen	BUN	mg/dL	8-25	8-25	6-23	6-23
Calcium	CA	g/dL	8.5-10.5	8.5-10.5	8.8-10.7	8.8-10.7
Chloride	CL	mmol/L	98-108	98-108	98-107	98-107
Carbon dioxide	CO2	mmol/L	23-32	23-32	22-31	22-31
Creatinine	CRE	mg/dL	0.6-1.5	0.6-1.5	0.5-1.2	0.5-1.2
Estimated glomerular filtration rate	eGFR	mL/min/1.73m^2	>60	>60	>60	>60
Glucose	GLU	mg/dL	70-110	70-110	70-110	70-110
Potassium	K	mmol/L	3.4-5.0	3.4-5.0	3.4-5.0	3.4-5.0
Sodium	NA	mmol/L	135-145	135-145	136-145	136-145

Supplementary Table 6 | Cohort sizes after exclusions

		Cohort for primary analysis (N)			Cohort for Fig 2 trajectories
Cohort	Initial No. of patients	No. with no repeat visits and stay ≥ 2 days	No. with ΔWBC ≥ 2	No. with 5+ day stay after WBC peak	No. of survivors
Limb amputation	1478	753	229	133	87
Colectomy	2571	1584	472	243	165
Cardiac surgery	4693	4693	2812	1836	1465
Cesarean section	15682	1273	612	49	39
Hip arthroplasty	9085	3249	496	142	100
Hysterectomy	2588	1049	237	68	51
Whipple surgery	1053	912	136	70	49
COVID-19	1686	1396	628	119	100
C. difficile colitis	634	383	117	54	46
Sepsis	6747	4730	1426	515	444
Myocardial Infarction	8132	6240	1262	656	327
Stroke	12889	2494	513	287	103

Legend: Exclusions are cumulative from left to right. WBC: white blood cell count.

Supplementary Table 7 | Comparison of mortality risk for cardiac surgery cohort stratified by direction percentiles, using interpolated and non-interpolated laboratory value trajectories

	With interpolation				Without interpolation			
	Day 3 direction percentile				Day 3 direction percentile			
	<50th	50-80	80-90	>90th	<50th	50-80	80-90	>90th
Mortality - %	0.60%	1.40%	4%	10%	1.20%	2.80%	3.00%	6.70%

	With interpolation				Without interpolation			
	Day 5 direction percentile				Day 5 direction percentile			
	<50th	50-80	80-90	>90th	<50th	50-80	80-90	>90th
Mortality - %	0.80%	1.30%	3%	16.40%	0.50%	1.60%	4.30%	11.50%

Supplementary Table 8 | Mortality or long stay risk for cardiac surgery cohort stratified by joint position and direction percentiles, using non-interpolated laboratory values

		Day 4 - Joint position and direction stratification					
		With interpolation			Without interpolation		
		Mortality or LOS > 14 - %			Mortality or LOS > 14 - %		
		Distance percentile			Distance percentile		
		<50th	50-80	>80th	<50th	50-80	>80th
Position percentile	<50th	1.60%	2.80%	14.30%	3.10%	5.50%	20%
	50-80	6.30%	6.80%	29.40%	8.30%	10%	28%
	>80th	22%	22.60%	52.80%	18.50%	20%	47.30%

Supplementary Table 9 | Adverse outcomes likelihood stratified by distance and position percentiles for various inflammatory cohorts.

			Surgery cohorts								
			Cardiac Surgery			Hip arthroplasty			Colectomy		
			Mortality or LOS > 14 - % (Number in sub-group)			Mortality or LOS > 10 - % (Number in sub-group)			Mortality or LOS > 14 - % (Number in sub-group)		
			Distance percentile			Distance percentile			Distance percentile		
			<50th	50-80	>80th	<50th	50-80	>80th	<50th	50-80	>80th
Day 1	Position percentile	<50th	3.9% (571)	8.6% (209)	1.8% (56)	1.6% (373)	2% (248)	5.8% (155)	6.6% (211)	10.2% (98)	8% (88)
		50-80	9.7% (259)	11.8% (93)	12.2% (49)	2.5% (241)	4.8% (146)	0% (81)	11.4% (123)	7.8% (51)	16.7% (48)
		>80th	26.9% (134)	25% (52)	39.1% (64)	14.6% (151)	10% (120)	6.3% (95)	47.7% (65)	33.3% (60)	31% (42)
Day 2	Position percentile	<50th	3.5% (367)	5.9% (236)	3.3% (183)	3% (302)	6.1% (197)	2.3% (86)	7% (213)	10.3% (107)	15.6% (45)
		50-80	10.2% (215)	15.9% (138)	11.3% (71)	2.2% (183)	3.6% (138)	4.7% (64)	11.7% (103)	11.3% (62)	18.4% (38)
		>80th	22.3% (103)	25.7% (101)	31% (71)	12.6% (87)	12.1% (107)	21.5% (65)	38.1% (63)	40.6% (32)	47.1% (51)
Day 3	Position percentile	<50th	1.4% (370)	1.9% (269)	10.5% (143)	6.5% (186)	3.5% (86)	19.4% (36)	7.1% (184)	5.4% (74)	13.8% (58)
		50-80	5.9% (188)	10.6% (104)	23.1% (91)	9.1% (77)	14.8% (54)	8% (25)	20.3% (59)	22.7% (66)	30.8% (26)
		>80th	12.8% (78)	27% (100)	43.6% (117)	12.1% (33)	32.4% (37)	29.4% (51)	18.5% (27)	29.3% (41)	71.4% (49)
Day 4	Position percentile	<50th	1.6% (378)	2.8% (216)	14.3% (70)	5% (101)	14.8% (61)	19.4% (31)	7.7% (130)	11.9% (67)	17% (53)
		50-80	6.3% (158)	6.8% (118)	29.4% (68)	4.7% (43)	23.3% (30)	25% (20)	20.4% (54)	29.8% (47)	34.8% (23)
		>80th	22% (59)	22.6% (62)	52.8% (127)	15.8% (19)	50% (32)	50% (30)	34.6% (26)	47.1% (34)	71.1% (38)
Day 5	Position percentile	<50th	3.3% (273)	7% (129)	11.6% (43)	8.3% (72)	31.3% (32)	40% (10)	12.5% (96)	19.1% (47)	18.8% (32)
		50-80	9.8% (123)	8.8% (80)	27.1% (59)	18.9% (37)	26.3% (19)	56.3% (16)	30.8% (52)	37% (46)	21.4% (28)
		>80th	34.9% (43)	31.8% (44)	62.1% (103)	41.2% (17)	58.3% (12)	40.9% (22)	66.7% (21)	53.3% (30)	80% (15)
Day 6	Position percentile	<50th	5.1% (197)	7.9% (76)	13.6% (44)	29.7% (37)	28% (25)	44.4% (9)	21.3% (61)	21.6% (37)	15.9% (44)
		50-80	14.9% (87)	25.4% (59)	37.5% (48)	32.3% (31)	56.3% (16)	38.5% (13)	33.3% (39)	50% (20)	39.3% (28)
		>80th	44.1% (34)	59.2% (49)	67.9% (56)	71.4% (14)	50% (6)	33.3% (6)	76.9% (26)	82.4% (17)	62.5% (16)

			Infection and ischemia cohorts								
			Myocardial infarction			COVID-19			Sepsis		
			Mortality - % (Number in sub-group)			Mortality - % (Number in sub-group)			Mortality - % (Number in sub-group)		
			Distance percentile			Distance percentile			Distance percentile		
			<50th	50-80	>80th	<50th	50-80	>80th	<50th	50-80	>80th
Day 1	Position percentile	<50th	5.2% (806)	4.9% (408)	5.6% (306)	4.7% (106)	5.6% (54)	6.5% (31)	8.1% (652)	5.3% (320)	17.3% (133)
		50-80	8% (439)	9.1% (263)	10.3% (175)	7.8% (64)	23.1% (13)	12.5% (16)	9% (333)	10% (219)	12.5% (120)
		>80th	18.8% (276)	17.1% (234)	21.3% (202)	25% (32)	26.1% (23)	25% (16)	20.3% (177)	12.8% (179)	13.1% (199)
Day 2	Position percentile	<50th	3.7% (758)	7.1% (520)	6% (250)	0.9% (111)	8.9% (45)	10.5% (19)	3.8% (559)	6% (418)	17.2% (145)
		50-80	9.3% (454)	8.8% (228)	7% (157)	10.2% (49)	17.4% (23)	10% (20)	8% (362)	12.7% (181)	21.7% (129)
		>80th	16.2% (370)	18.6% (194)	24.3% (181)	22.7% (22)	9.1% (11)	52% (25)	15.3% (229)	21.1% (133)	16.2% (185)
Day 3	Position percentile	<50th	6.2% (616)	6.5% (401)	4.5% (178)	5.5% (91)	0% (34)	5.3% (38)	3.8% (522)	7.9% (341)	14.3% (133)
		50-80	8.4% (381)	9.5% (168)	11% (154)	3.4% (29)	20% (20)	26.3% (19)	7.7% (287)	7.4% (162)	15% (140)
		>80th	20.2% (267)	19% (126)	24% (171)	23.1% (13)	38.5% (13)	43.5% (23)	12.4% (186)	15.5% (97)	27.6% (181)
Day 4	Position percentile	<50th	5.8% (521)	8.4% (250)	6.2% (162)	4.3% (92)	6.1% (33)	2.9% (34)	3.2% (467)	5.8% (224)	11.1% (126)
		50-80	9.7% (300)	11.8% (161)	10.8% (120)	16.7% (24)	12.5% (16)	17.6% (17)	5.8% (259)	13% (193)	21.7% (115)
		>80th	17.7% (175)	24.7% (182)	28.1% (128)	28.6% (14)	40% (10)	40% (10)	10.2% (128)	17.2% (122)	29.4% (153)
Day 5	Position percentile	<50th	6.9% (348)	7% (273)	10.3% (155)	3% (67)	4.3% (47)	0% (15)	3.4% (417)	5.8% (172)	14.5% (117)
		50-80	13.6% (221)	8.2% (195)	8.8% (113)	20% (20)	0% (16)	35.3% (17)	8.4% (238)	14.8% (176)	14.9% (121)
		>80th	23% (200)	27.4% (95)	35.2% (91)	26.7% (15)	20% (15)	44.4% (9)	18.3% (104)	17.3% (139)	20.3% (74)
Day 6	Position percentile	<50th	8.7% (343)	8% (238)	6.3% (112)	1.5% (66)	0% (16)	9.5% (21)	2.8% (393)	5.6% (126)	13.8% (87)
		50-80	11.5% (218)	13.6% (154)	11.1% (72)	4.2% (24)	17.6% (17)	6.7% (15)	7.1% (198)	17% (147)	22.2% (90)
		>80th	27.2% (180)	24.6% (65)	25.8% (66)	12.5% (8)	57.1% (14)	50% (10)	11.7% (77)	19.5% (128)	21.5% (79)

Legend: LOS: length of hospital stay. LOS is from admission for ischemia and infection cohorts, and from time of operation for surgical cohorts.

Supplementary References

1. Shahian, D. M. *et al.* The Society of Thoracic Surgeons 2018 Adult Cardiac Surgery Risk Models: Part 1—Background, Design Considerations, and Model Development. *Annals of Thoracic Surgery* (2018) doi:10.1016/j.athoracsur.2018.03.002.

2. O'Brien, S. M. *et al.* The Society of Thoracic Surgeons 2018 Adult Cardiac Surgery Risk Models: Part 2—Statistical Methods and Results. *Annals of Thoracic Surgery* (2018) doi:10.1016/j.athoracsur.2018.03.003.
