# Supplementary material for: Human acute inflammatory recovery is defined by co-regulatory dynamics of white blood cell and platelet populations
Source: Nat Commun. 2022 Aug 22;13:4705. doi: 10.1038/s41467-022-32222-2 (PMC9395541; doi:10.1038/s41467-022-32222-2)
Supplement: Supplementary file 3 — Description of Additional Supplementary Files [file 41467_2022_32222_MOESM3_ESM.pdf]

Description of Additional Supplementary Files

Supplementary Movie 1 | Cardiac surgery WBC-PLT trajectories stratified by pre-operative laboratory values. The movie illustrates the evolution of the four trajectories in Fig. 1c, iv from pre-operative to day 7 post-operation. Each trajectory gives the mean WBC-PLT values over time for patients with favorable outcomes (stay < 14 days, survivor). Patients are separated into four quadrants based on whether their pre-operative WBC and PLT values were above or below the median.

Supplementary Software 1 | A series of MATLAB files designed to generate the primary results in this manuscript for a sample dataset. Steps for appropriate implementation of the software are given in the file README.pdf
