# Supplementary material for: Human acute inflammatory recovery is defined by co-regulatory dynamics of white blood cell and platelet populations
Source: Nat Commun. 2022 Aug 22;13:4705. doi: 10.1038/s41467-022-32222-2 (PMC9395541; doi:10.1038/s41467-022-32222-2)
Supplement: Supplementary file 5 — Supplementary Software 1 [file 41467_2022_32222_MOESM5_ESM.zip › Supplementary software/README FILE.pdf]

README FILE

README file for example code associated with Nature Communications manuscript: *Human Acute Inflammatory Recovery is Defined by Co-Regulatory Dynamics of White Blood Cell and Platelet Populations*.

All code was written and commented by Dr Brody H Foy.
Contact email: bfoy1@mgh.harvard.edu
Website: www.brodyfoy.com/

DISCLAIMER
The code base and associated dataset are intended for illustrative purposes only. They are meant as an aid to better understand the scientific content of the associated manuscript and should not be used with any dataset other than the associated illustrative dataset ("Toy Dataset.xlsx"). Any results generated from this dataset and code base are illustrative only, and no guarantee is made for accuracy of the associated code base. This code is provided without warranty, implied or otherwise.

The code base is a translated version of the code base used in the associated study, rewritten with emphasis on user readability. As such, if any errors exist in this code they do not necessarily imply an equivalent error in the manuscript results. Please contact Dr Foy if you believe there is an error in this code base that may have ramifications for the associated manuscripts major findings.

This program is free and can be modified/redistributed in any way under the terms of the GNU General Public License version 3, as published by the Free Software Foundation. It is distributed without warranty implied or otherwise, or any implication of merchantability or fitness for a particular purpose. A copy of the GNU General Public License can be found at: http://www.gnu.org/licenses/

CODE DESCRIPTION
This set of MATLAB function and script files is designed to illustrate how key figures and results within the associated manuscript were generated. Collectively, this illustrates 5 functions:

- Generate_PhasePlane_Curve.m : This function generates a WBC-PLT phase-plane curve, similar to that within **Fig. 1c, Fig. 2a, and Fig. 2c** of the manuscript.
- Generate_Position_Plot.m : This function generates a position percentile reference plot, similar to that within **Fig. 4a**
- Generate_Direction_Plot.m : This function generates a direction percentile reference plot, similar to that within **Fig. 1d**, and **Fig. 4a**.
- Generate_ModelFits.m : This function fits WBC and PLT models (as detailed in the manuscript) to given data, and plots the associated data and fits. The plots produced are similar to those within **Fig. 2b, Fig. 2d,** and **Fig. 3** of the manuscript.
- Generate_RiskRatio_Plot.m : This function generates position-direction risk plots, and calculates associated risk ratios, similar to those within **Fig. 4b-c** of the manuscript.

All of these functions are called in succession by the primary script file **MAIN_FILE.m**.

DATASET
To illustrate these functions, an artificial dataset of 100 surgery cohort patients has been created. This

artificial dataset contains WBC and PLT data for the 100 patients, interpolated and evenly sampled every 12hrs from immediately post-operation to 20 days later, alongside a pre-operative value. Patients who discharge before 20 days will have missing data for any time points post discharge.

Alongside this, two outcomes are given for each patient:

- Mortality: a logical denoting whether they died during their stay (or within 30 days post discharge). 1 denotes deceased, and 0 denotes survived.
- LengthOfHospitalStay: The length of time from end of surgery to hospital discharge, in days.

This dataset was generated by randomly selected 100 patients from the cardiac surgery cohort in the associated manuscript, with 90 survivors, and 10 deceased. Patient data has been anonymized according to PHI restrictions.

Given the significant reduction in size of this dataset comparative to cohorts in the manuscript, key results may not be perfectly replicated by the code functions. The dataset and code are meant to be illustrative only.

RUN ENVIRONMENT AND INSTRUCTIONS

To run the code base, execute script file MAIN_FILE.m, while having all associated script files and "Toy Dataset.xlsx" in the same directory. Do not change the name of any file, including the dataset.

This software was tested on a Dell XPS 17 laptop running a 64-bit Windows operating system, with 64-GB RAM, and an 11th Gen Intel i9-11900 H 2.5GHz processor. MATLAB version 2021b (64-bit) was used, with no additional toolboxes. On this machine, the average code execution time was 0.8s.

Running this file produced the following text output:

```
PLT model fit:

      a: 3.7769

      b: 11.3226

      c: 0.1689

    Rsq: 0.9401


WBC model fit:

      a: 55.5893

      b: 33.2318

    tau: 2.5000

    Rsq: 0.9971


Risk ratio:
```

10 (0 - 30)

Running this file also produced 5 figures (included in order):

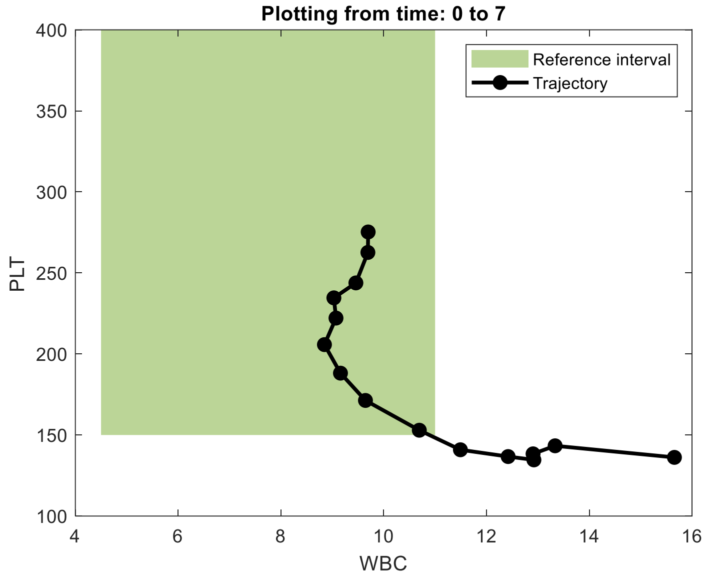

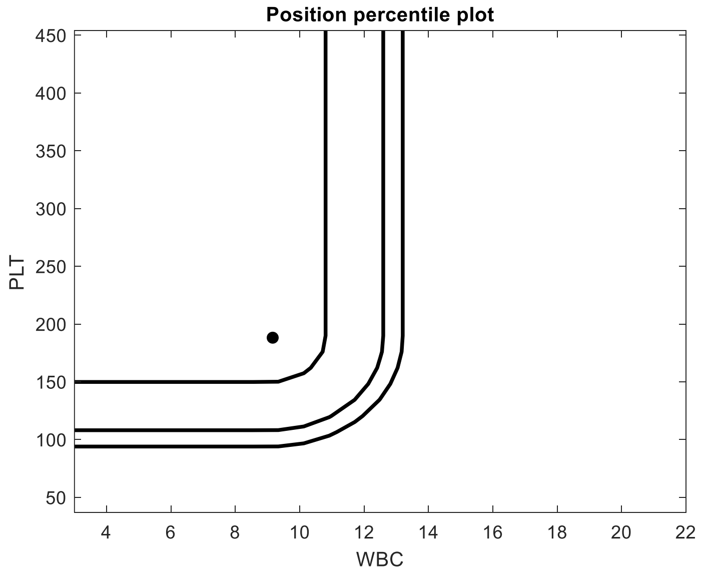

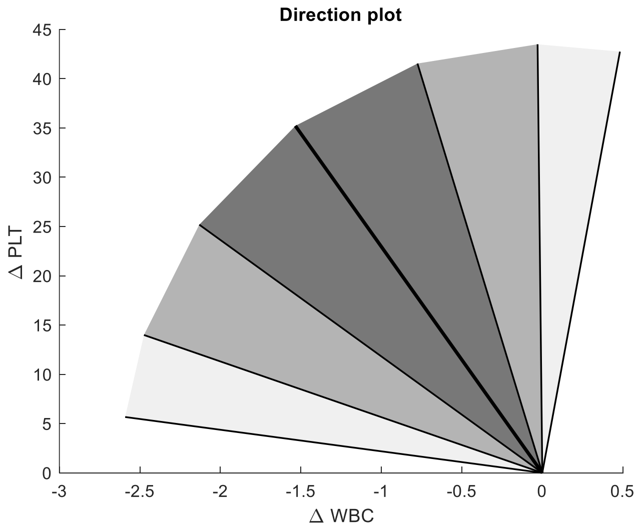
Direction plot
Δ PLT
Δ WBC
45
40
35
30
25
20
15
10
5
0
-3
-2.5
-2
-1.5
-1
-0.5
0
0.5

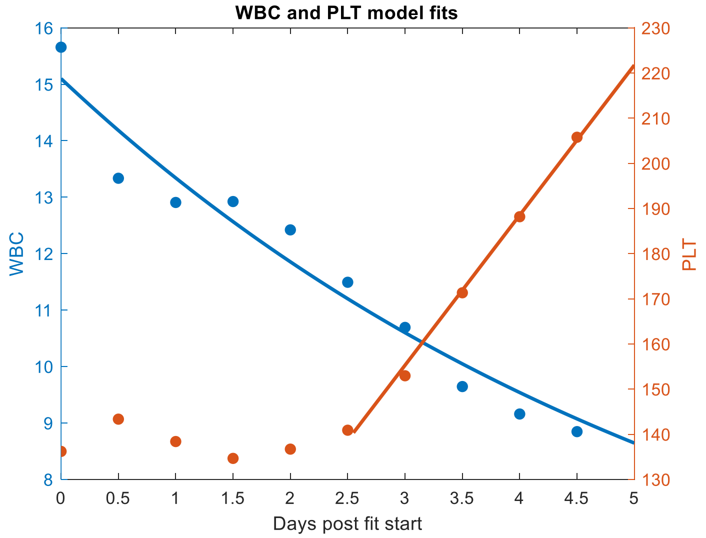
WBC and PLT model fits
WBC
PLT
Days post fit start
16
15
14
13
12
11
10
9
8
230
220
210
200
190
180
170
160
150
140
130
0
0.5
1
1.5
2
2.5
3
3.5
4
4.5
5

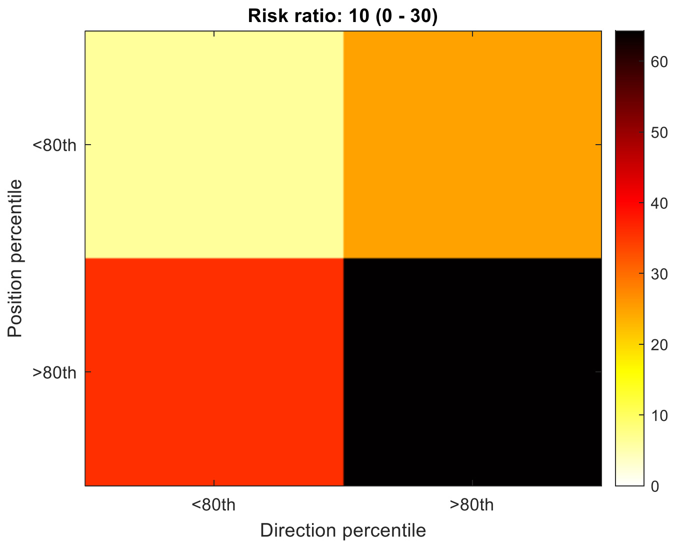
Risk ratio: 10 (0 - 30)
Position percentile
<80th
>80th
Direction percentile
<80th
>80th
60
50
40
30
20
10
0
