# Supplementary material for: Human acute inflammatory recovery is defined by co-regulatory dynamics of white blood cell and platelet populations
Source: Nat Commun. 2022 Aug 22;13:4705. doi: 10.1038/s41467-022-32222-2 (PMC9395541; doi:10.1038/s41467-022-32222-2)
Supplement: Supplementary file 6 — Reporting Summary [file 41467_2022_32222_MOESM6_ESM.pdf]

nature portfolio

Corresponding author(s): John Higgins

Last updated by author(s): Jul 3, 2022

Reporting Summary

Nature Portfolio wishes to improve the reproducibility of the work that we publish. This form provides structure for consistency and transparency in reporting. For further information on Nature Portfolio policies, see our Editorial Policies and the Editorial Policy Checklist.

Statistics

For all statistical analyses, confirm that the following items are present in the figure legend, table legend, main text, or Methods section.

n/a	Confirmed	
☐	☒	The exact sample size (n) for each experimental group/condition, given as a discrete number and unit of measurement
☐	☒	A statement on whether measurements were taken from distinct samples or whether the same sample was measured repeatedly
☐	☒	The statistical test(s) used AND whether they are one- or two-sided<br>*Only common tests should be described solely by name; describe more complex techniques in the Methods section.*
☐	☒	A description of all covariates tested
☐	☒	A description of any assumptions or corrections, such as tests of normality and adjustment for multiple comparisons
☐	☒	A full description of the statistical parameters including central tendency (e.g. means) or other basic estimates (e.g. regression coefficient) AND variation (e.g. standard deviation) or associated estimates of uncertainty (e.g. confidence intervals)
☐	☒	For null hypothesis testing, the test statistic (e.g. F, t, r) with confidence intervals, effect sizes, degrees of freedom and P value noted<br>*Give P values as exact values whenever suitable.*
☒	☐	For Bayesian analysis, information on the choice of priors and Markov chain Monte Carlo settings
☒	☐	For hierarchical and complex designs, identification of the appropriate level for tests and full reporting of outcomes
☐	☒	Estimates of effect sizes (e.g. Cohen's d, Pearson's r), indicating how they were calculated

Our web collection on statistics for biologists contains articles on many of the points above.

Software and code

Policy information about availability of computer code

Data collection	All data within this study was collected using the Partners Healthcare Research Patient Data Registry and Electronic Data Warehouse. Data from EDW was collected from a database using custom SQL queries, via a Windows 2014 MS SQL interface. Data from RPDR was collected using the GUI interface that this program provides.
Data analysis	All analysis within this study was performed using MATLAB version 2021a. Code (and associated documentation) for replicating the primary results in a reduced dataset (due to PHI constraints) has been provided as part of the Supplementary Materials associated with this submission. We have chosen to provide code this way instead of through a public repository as the code is primarily intended to illustrate how the primary results in the manuscript were generated, and is not intended as a living piece of software.

For manuscripts utilizing custom algorithms or software that are central to the research but not yet described in published literature, software must be made available to editors and reviewers. We strongly encourage code deposition in a community repository (e.g. GitHub). See the Nature Portfolio guidelines for submitting code & software for further information.

Data

Policy information about availability of data

All manuscripts must include a data availability statement. This statement should provide the following information, where applicable:

- Accession codes, unique identifiers, or web links for publicly available datasets
- A description of any restrictions on data availability
- For clinical datasets or third party data, please ensure that the statement adheres to our policy

Source data for all figures are provided with this paper, as part of the Supplementary Information/Source Data files. Some of the detailed raw data analyzed collected in this study cannot be made available due to IRB restrictions on the sharing of protected health information. Consistent with this requirement, a sample

dataset has also been included in the Supplementary Information, alongside custom code for generating equivalents of the primary figures/results using the artificial dataset.

Field-specific reporting

Please select the one below that is the best fit for your research. If you are not sure, read the appropriate sections before making your selection.

[x] Life sciences [ ] Behavioural & social sciences [ ] Ecological, evolutionary & environmental sciences

For a reference copy of the document with all sections, see nature.com/documents/nr-reporting-summary-flat.pdf

Life sciences study design

All studies must disclose on these points even when the disclosure is negative.

Sample size	Sample size was limited by availability/frequency of the clinical populations during the time period of this study (2016-2020). 2016 was chosen as the lower bounds of data collection, due to limitations in data availability before this point. Of the 12 cohorts analyzed, after exclusions (detailed below) the minimum cohort size was 383, and 9/12 cohorts had >1000 patients, sufficient to power our analyses.
Data exclusions	Data exclusions are explained in detail in the manuscript and supplementary materials. In short, patients were excluded if they were under 18yrs old, had a less than 48hr hospital stay, or did not have at least 1 blood count and metabolic panel during their stay. These criteria were chosen in advance to limit to adults, and to ensure sufficient data availability for each patient. Certain clinical cohorts had exclusion criteria specific to the clinical setting (e.g. laparoscopic surgeries were excluded, due to not typically producing significant inflammatory responses). These criteria are detailed in depth in the supplementary materials, and were all defined prior to data collection.
Replication	All clinical cohorts were split into exploratory and validation cohorts. All exploratory analysis was performed solely in the exploratory cohorts, and validation cohorts were not analyzed until the primary figures of the manuscript had been fully created/outlined. To ensure robustness, all estimates of outcomes were performed in the validation cohorts, using thresholds defined in the exploratory cohorts, except in a few of the smaller clinical cohorts. In these smaller cohorts, outcomes were calculated across the entire cohort, and are clearly noted as such in the manuscript. All attempts at replication were successful.
Randomization	This is not relevant to our study, given the study was retrospective and observational. For splitting into exploratory and validation cohorts, splits were performed based on admission year, instead of randomization, to allow for detection of any possible time-dependent effects between the cohorts.
Blinding	Blinding was not relevant to our study, given the retrospective and observational nature.

Reporting for specific materials, systems and methods

We require information from authors about some types of materials, experimental systems and methods used in many studies. Here, indicate whether each material, system or method listed is relevant to your study. If you are not sure if a list item applies to your research, read the appropriate section before selecting a response.

Materials & experimental systems

n/a	Involved in the study	
[x]	[ ]	Antibodies
[x]	[ ]	Eukaryotic cell lines
[x]	[ ]	Palaeontology and archaeology
[x]	[ ]	Animals and other organisms
[ ]	[x]	Human research participants
[x]	[ ]	Clinical data
[x]	[ ]	Dual use research of concern

Methods

n/a	Involved in the study	
[x]	[ ]	ChIP-seq
[x]	[ ]	Flow cytometry
[x]	[ ]	MRI-based neuroimaging

Human research participants

Policy information about studies involving human research participants

Population characteristics	For each of the procedure/diagnosis cohorts in this study, summary characteristics (N, age, gender, key laboratory results, outcomes) are presented in detail in Table 1. For the cardiac surgery cohort - the primary cohort in this study - more expansive sets of demographics, laboratory measures, pre-operative risk factors, and post-operative outcomes are presented in Supplementary Table 1. For the cardiac surgery cohort, mean age was 64yrs, gender was 71% male, race was 86% white/ caucasian, and 30-day mortality was 2.3%.
Recruitment	This study involved analysis of only retrospective data. No active recruitment of patients occurred. All patients with the recorded diagnosis/procedure were collected from the database from 2016-2019 (for the cardiac surgery cohort) and 2016-2020 (all other cohorts). 2016 was chosen as the start year for analysis due to a shift in MGB database infrastructure

prior to this point, meaning earlier results were not consistently available. Cardiac surgery cohort analysis occurred primarily in 2020, and all other cohort analysis occurred primarily in 2021, leading to different end dates for data collation. Analysis was primarily limited to each patient's inpatient course, meaning detailed patient data was available, and there was no bias due to patient dropout. Data was also analysed from a second hospital (BWH, details in manuscript) to help mitigate potential bias due to differences across medical centers. All results have been carefully framed in context of the inherent limitations of retrospective studies.

Ethics oversight	The study was performed in compliance with a research protocol approved by the local institutional review board (IRB). The Mass General Brigham IRB approved access to patient identifiers for the purpose of linking different sources of patient data during the analysis phase of the project, with links to patient identifiers to be destroyed at the conclusion of the project. The IRB approved publication of only summary patient data or limited individual de-identified patient data. The IRB considered the risks to research subjects, the selection of subjects, the privacy of subjects, and confidentiality of the data and determined that the study's data management procedures and publication plans justified an authorization for a waiver of informed consent.

Note that full information on the approval of the study protocol must also be provided in the manuscript.
